# Genetic, Epigenetic, Genomic and Microbial Approaches to Enhance Salt Tolerance of Plants: A Comprehensive Review

**DOI:** 10.3390/biology10121255

**Published:** 2021-12-01

**Authors:** Gargi Prasad Saradadevi, Debajit Das, Satendra K. Mangrauthia, Sridev Mohapatra, Channakeshavaiah Chikkaputtaiah, Manish Roorkiwal, Manish Solanki, Raman Meenakshi Sundaram, Neeraja N. Chirravuri, Akshay S. Sakhare, Suneetha Kota, Rajeev K. Varshney, Gireesha Mohannath

**Affiliations:** 1 Department of Biological Sciences, Birla Institute of Technology and Science, Pilani, Hyderabad Campus, Hyderabad 500078, India; p20170002@hyderabad.bits-pilani.ac.in (G.P.S.); sridev.mohapatra@hyderabad.bits-pilani.ac.in (S.M.); 2 Biological Sciences and Technology Division, CSIR-North East Institute of Science and Technology (CSIR-NEIST), Jorhat 785006, India; debajitbnc@gmail.com (D.D.); channakeshav@neist.res.in (C.C.); 3 ICAR-Indian Institute of Rice Research, Hyderabad 500030, India; Satendra.KM@icar.gov.in (S.K.M.); phosphodiester21@gmail.com (M.S.); rmsundaram34@gmail.com (R.M.S.); cnneeraja@gmail.com (N.N.C.); sakhare.akshaya@gmail.com (A.S.S.); 4 Center of Excellence in Genomics & Systems Biology, International Crops Research Institute for the Semi-Arid Tropics (ICRISAT), Hyderabad 502324, India; manishroorkiwal@gmail.com; 5 The UWA Institute of Agriculture, The University of Western Australia, Perth, WA 6009, Australia; 6 State Agricultural Biotechnology Centre, Centre for Crop and Food Innovation, Food Futures Institute, Murdoch University, Murdoch, WA 6150, Australia

**Keywords:** salinity stress, genomics breeding, epiRIL, genome editing, plant growth-promoting rhizobacteria, genetic engineering

## Abstract

**Simple Summary:**

Globally, soil salinity, which refers to salt-affected soils, is increasing due to various environmental factors and human activities. Soil salinity poses one of the most serious challenges in the field of agriculture as it significantly reduces the growth and yield of crop plants, both quantitatively and qualitatively. Over the last few decades, several studies have been carried out to understand plant biology in response to soil salinity stress with a major emphasis on genetic and other hereditary components. Based on the outcome of these studies, several approaches are being followed to enhance plants’ ability to tolerate salt stress while still maintaining reasonable levels of crop yields. In this manuscript, we comprehensively list and discuss various biological approaches being followed and, based on the recent advances in the field of molecular biology, we propose some new approaches to improve salinity tolerance of crop plants. The global scientific community can make use of this information for the betterment of crop plants. This review also highlights the importance of maintaining global soil health to prevent several crop plant losses.

**Abstract:**

Globally, soil salinity has been on the rise owing to various factors that are both human and environmental. The abiotic stress caused by soil salinity has become one of the most damaging abiotic stresses faced by crop plants, resulting in significant yield losses. Salt stress induces physiological and morphological modifications in plants as a result of significant changes in gene expression patterns and signal transduction cascades. In this comprehensive review, with a major focus on recent advances in the field of plant molecular biology, we discuss several approaches to enhance salinity tolerance in plants comprising various classical and advanced genetic and genetic engineering approaches, genomics and genome editing technologies, and plant growth-promoting rhizobacteria (PGPR)-based approaches. Furthermore, based on recent advances in the field of epigenetics, we propose novel approaches to create and exploit heritable genome-wide epigenetic variation in crop plants to enhance salinity tolerance. Specifically, we describe the concepts and the underlying principles of epigenetic recombinant inbred lines (epiRILs) and other epigenetic variants and methods to generate them. The proposed epigenetic approaches also have the potential to create additional genetic variation by modulating meiotic crossover frequency.

## 1. Introduction

Across the world, soil salinization has been on the rise due to factors such as sea-level rise owing to global warming, overexploitation of coastal groundwater aquifers causing seawater intrusion, depletion of groundwater table, drought, usage of poor-quality groundwater for irrigation, inappropriate irrigation practices, poor drainage, improper usage of fertilizers and pesticides, and various other human activities. Globally, soil salinity is one of the most destructive abiotic stresses faced by crop plants resulting in significant yield losses to a magnitude of ~10% of global output. Soil salinity can also degrade arable soils, which are heavily irrigated [1]. Salt-affected soils are relatively widespread in arid and semi-arid climates compared to humid regions. Worldwide, soil salinity affects about 20% of arable land and 50% of irrigated land [2]. Salt stress drastically reduces agricultural productivity owing to its adverse impacts on all aspects of plant development, including seed germination, vegetative growth, flowering, and seed set. [3,4,5,6]. It also affects plants’ ability to fix biological nitrogen [7]. Reduced crop growth and yield are typically caused by salinity-induced water stress, oxidative stress, ion toxicity, ionic and nutritional imbalances, membrane disorganization, reduced cell division and expansion, and disruption of key metabolic processes. Several past studies at large had focused on understanding the damages caused by salinity stress on the crop plants and attempts have been made to breed or engineer plants to counter salt stress effectively [8].

Conventional plant breeding methods have proven to be one of the best strategies to improve salinity tolerance of crop plants, but such methods are more laborious, time-consuming, and depend on access to germplasm containing sufficient genetic variability [9]. In recent years, genomics-assisted breeding approaches, including marker-assisted breeding, have been employed to make the breeding process less time-consuming [10,11]. In parallel, genetic engineering involving selective modulation of expression of one or few transgenes has been frequently used to understand the mechanisms underlying various stress responses. Recent advances in DNA/RNA sequencing and bioinformatics have made whole-genome sequencing a regular feature of current molecular breeding modules. Genomics can complement marker-assisted breeding and genetic engineering in rapidly identifying genes/QTL associated with desirable traits and their introduction into favorable genotypes [11]. The invention of genome editing technology has added one more dimension to genetic engineering, and since its introduction, it has become a valuable tool to knock-down/knock-out/knock-in genes of interest [12].

Soil health, a measure of soil fertility and soil quality, also refers to the prolonged ability of soil to function as a vital living ecosystem that sustains plants, animals, and humans [13]. Increased salinity negatively impacts soil health and is a serious environmental issue [14]. Beneficial soil microbes that are part of the soil microbiome are known to colonize the rhizosphere of plants and aid in the maintenance of soil health [15]. Several studies have been carried out to understand the role of plant growth-promoting rhizobacteria (PGPR) in alleviating abiotic stress, including salt stress. In this comprehensive review, we summarize the latest findings from several studies which employed genetic, genomic, molecular, and PGPR-based approaches to enhance salt tolerance of crop plants. Lastly, we elaborate on the concept and methods to generate epigenetic recombinant inbred lines (epiRILs) and other novel epigenetic variants, which can be screened to identify novel epialleles or epiQTLs that can impart enhanced salt stress tolerance to plants.

## 2. Physiological and Biochemical Basis of Salt Tolerance

Several mechanisms underlying the physiological and biochemical basis of salt tolerance have been elucidated. Under soil salinity conditions, plants experience hyperosmotic stress first and then hyper-ionic stress [16,17], including ionic and oxidative stress. Hyperosmotic stress occurs due to a decrease in the water absorption capacity of plant roots caused by a decrease in the water potential in the soil [18]. Subsequently, water loss from leaves accelerated by osmotic stress leads to the accumulation of ions, causing hyperionic stress or ion toxicity [19]. Under high saline conditions, accumulated salts, mostly the excess Na^+^ and Cl^−^ in the cells, cause osmotic stress, and the resulting ion toxicity interferes with several physiological and biochemical processes. Major physiological and biochemical mechanisms for survival in high salt-affected soils include modulation of ion uptake and transport, ion homeostasis and compartmentalization, synthesis of osmoprotectants and antioxidant compounds, regulation of hormones during salt stress, and activation of stress signaling pathways.

### 2.1. Modulation of Ion Uptake and Transport

Plants have developed effective sensory mechanisms to recognize stress conditions. Soil-based salt stress is first sensed by roots that minimize the entry of salts into the xylem, a phenomenon called ‘salt exclusion’ [20,21]. Most plants exclude about 98% of the salt in the soil solution permitting only ~2% to be transported to the shoot through the xylem, but this number is higher for salt-sensitive plant species (e.g., rice allows ~6% of salts) [18,22]. Ion uptake and the transport of salts in plants occur through apoplastic and symplastic transport pathways. Once the salt enters the plant, it accumulates at different concentrations in different plant organs. Shoots are the major plant parts where salts are accumulated. The older leaves accumulate higher concentrations of Na^+^ (and Cl^−^) within shoots than younger leaves [23,24].

### 2.2. Ion Homeostasis and Compartmentalization

Ion homeostasis refers to the maintenance of ion concentrations across plant cells. Plants maintain ion homeostasis in the cytosol by various mechanisms such as balancing ion uptake through the efficient regulation of influx and efflux of salts, sequestration of excess salts into the vacuole, and salt compartmentalization in older tissues to minimize salt injury for younger tissues. Tissue tolerance is the ability of tissues to tolerate accumulated salts (Na^+^ and Cl^−^), and, presumably, it reflects the ability of tissues to compartmentalize toxic ions and maintain Na^+^ and Cl^−^ concentrations as low as 10–30 mM within the cytoplasm [25]. The highest tolerable concentration has been estimated to be 50 to 100 mM, beyond which many enzymes lose their activities [19,26]. Estimating tissue tolerance is a tedious process, and that the identification of a molecular marker associated with this trait would significantly speed up the breeding efforts for salt tolerance.

How ion homeostasis contributes to salt tolerance through activation of stress sensing and signaling pathways has been well-studied. Plant salt tolerance involves several endosomal transport proteins, regulation of organellar pH, and ion homeostasis [27,28,29]. The major ions involved in the salt stress signaling include Na^+^, K^+^, H^+^, and Ca^2+^, and their interplay brings homeostasis in the cell. Largely, ion homeostasis is governed by the proton pumps and other ion transporters located on the plasma membrane and tonoplast (the membrane surrounding the vacuoles) and their associated components such as ATPases and pyrophosphatases. Plasma membrane-located transporters belonging to the histidine kinase transporter (HKT) family play a vital role in salt tolerance by regulating levels of Na^+^, K^+^, and root-to-shoot Na^+^ partitioning. Several HKT genes have been characterized by different plant species [30], and are categorized into class I and II types. Class I HKT transporters mediate selective Na^+^ transport [31,32], while Class II HKT transporters are involved in Na^+^–K^+^ co-transport [33].

Various carrier proteins, channel proteins, antiporters, symporters, and ion channels play a critical role in maintaining ion gradients across the cell membrane, which are vital for regulating the activity of enzymes involved in various physiological and cellular processes. Low Na^+^ in the cytoplasm is maintained majorly by the tonoplast-localized Na^+^/H^+^ exchanger (NHX) and the plasma membrane-localized Na^+^/H^+^ antiporter SALT OVERLY SENSITIVE 1 (SOS1, also known as NHX7). Mostly, NHXs are essential for Na^+^ detoxification via sequestration of Na^+^ within the vacuole, while the SOS signaling pathway regulates the efflux of Na^+^ ions out of the cell. Tonoplast has two types of H^+^ pumps; vacuolar type H^+^-ATPase (V-ATPase) and vacuolar pyrophosphatase (V-PPase) [34], of which V-PPase is more prevalent. Maintenance of stable K^+^ acquisition and distribution in plant cells is important in balancing the toxic effects of Na^+^ accumulation. Net K^+^ selective influx and K^+^ efflux are maintained by inward-rectifying and out-ward-rectifying K^+^ channels, respectively [35]. NHX-type proteins play a key role in compartmentalizing K^+^ into vacuoles and maintaining cellular pH homeostasis [36].

### 2.3. Synthesis of Osmoprotectants and Antioxidant Compounds

Plants adapt to osmotic stress by synthesizing compatible osmolytes, which lower intracellular osmotic potential and thus facilitate water uptake and simultaneously prevent water loss. They maintain cell integrity and protect the structure of cells. Osmoprotectants, synthesized in the cytoplasm, are small organic molecules with a neutral charge and low toxicity even at higher concentrations, and they protect cells from osmotic stress. Also known as compatible solutes, they balance the osmotic difference between the cytosol and vacuoles or between adjacent cells. Osmolytes constitute amino acids and their derivatives (e.g., proline, glycine betaine), organic acids (e.g., salicylate, citrate, malate, malonate γ-amino butyric acid), reducing and non-reducing sugars/carbohydrates (e.g., sucrose, fructose, glucose, trehalose, raffinose, and fructans), sugar alcohols and polyols (e.g., pinitol, cyclitol, mannitol, sorbitol, myo-inositol), polyamines (e.g., putrescine, spermidine, spermine), quaternary ammonium compounds (e.g., β-alanine-betaine, proline-betaine, hydroxyprolinebetaine) and some proteins. The accumulation of osmolytes or compatible solutes in higher concentrations in cytosol has been associated with counteracting deleterious effects of salinity stress [19,37].

### 2.4. Regulation of Hormones during Salt Stress

Besides their vital role in plant growth and development, plant hormones also mediate plant adaptation to several abiotic stresses. In response to salt stress, plants activate the defense pathways through synthesis, signaling, and metabolism of stress response- and plant growth-promoting hormones. The crosstalk among various hormones mediates stress tolerance. Among the well-characterized hormones, abscisic acid (ABA), ethylene, salicylic acid (SA), and jasmonic acid (JA) are regarded as stress response hormones, while auxin, gibberellic acid (GA), cytokinins, brassinosteroids, and strigolactones are categorized as growth-promoting hormones. ABA plays a vital role in response to salt stress response, which activates the genes involved in ABA biosynthesis induced by calcium-dependent phosphorylation cascade and their downstream signaling events. Indeed, high levels of endogenous ABA were observed in rice, brassica, and maize when grown under salt stress [38]. In response to salt-induced osmotic stress, ABA regulates the stomatal opening and closing for osmotic adjustment. ABA integrates several complex developmental processes and adaptive signaling pathways, including activation of sucrose non-fermenting 1-related protein kinases (SnRK2s), which further regulate osmotic homeostasis [39]. ABA-mediated signaling also upregulates several genes belonging to the mitogen-activated protein kinase (MAPKs) family, Ca^2+^-related kinases, and stress-responsive transcription factors. Among other hormones, JA is involved in the salt-induced inhibition of primary root growth, and auxin regulates plant growth adaptive mechanism through salt-inhibited root growth plasticity via crosstalk with ABA [40]. High salt stress causes excessive accumulation of ABA, disrupting the distribution of auxin and lateral root development [41]. On the other hand, cytokinins act as negative regulators of salinity tolerance, and accordingly, salt stress results in decreased endogenous levels of cytokinins and increased levels of ABA [42,43]. Brassinosteroids regulate plant salt tolerance by interacting with other plant hormones and by playing a key role in ROS scavenging [44]. Overall, different plant hormones act differently under salinity stress and collectively control the balance between growth and salt stress responses [41].

### 2.5. Activation of Stress-Signaling Pathways

To adapt to salt stress, plants activate ionic and osmotic signaling pathways among which, the Salt Overly Sensitive (SOS) signaling pathway is of major importance [45]. The SOS pathway consists of three key proteins named SOS1 (Na^+^ efflux-regulating plasma membrane Na^+^/H^+^ antiporter), SOS2 (a serine/threonine kinase), and SOS3 (a myristoylated calcium-binding protein), combinedly constituting a signaling system that maintains ionic homeostasis [46,47,48]. SOS1 gene encodes a plasma membrane Na^+^/H^+^ antiporter that regulates Na^+^ efflux at the cellular level and long-distance Na^+^ transport from shoot to the root [48]. SOS2 encodes a serine/threonine kinase, which is activated by salt stress-induced Ca^+^ signals [46]. SOS3 encodes a myristoylated calcium-binding protein that appears to function as a primary calcium sensor to perceive enhanced cytosolic Ca^2+^ triggered by an excess of cytoplasmic Na^+^ [47]. The interaction between SOS2 and SOS3 proteins activates upstream kinases which then phosphorylate SOS1 protein [49,50]. The phosphorylated SOS1 reduces Na^+^ toxicity by increasing Na^+^ efflux from the cytoplasm to the apoplast. Likewise, the involvement of MAP kinase cascades and ABA-dependent SnRK2-mediated signaling in response to salt stress has been demonstrated in Arabidopsis [51,52], alfalfa [53], and rice [54].

## 3. The Genetic Basis of Tolerance to Salinity in Plants

The development of salt-tolerant varieties using conventional and/or modern breeding approaches has been vital in effectively managing salt stress. Akin to improving other traits, efforts to improve salt stress tolerance have largely utilized genetic variation available among the germplasm, including the landraces [55]. Typically, the genotypes that offer a better response to salt stress also possess multiple undesirable traits, and therefore, introgression of desirable genes from donors into otherwise high-yielding varieties has been employed through classical breeding programs [56]. For example, traditional cultivars and landraces, which are naturally tolerant to salt stress are available but possess undesirable traits such as photosensitivity, tall plant type prone to lodging, low yield, and poor grain quality [57,58]. Breeding for salinity tolerance dates back to the 1970s, wherein attempts were made to exploit genetic variation available in the traditional landraces [59,60]. However, progress has been slow due to the complex and polygenic nature of the trait involving several physiological mechanisms [61]. It is a typical tradeoff between yield and stress tolerance attributes of plants because some traits are mutually exclusive. Initially, attempts were made to understand the genetic basis of salt tolerance in legume and cereal crops [62,63,64,65,66,67]. Genetic and biostatistical studies on rice identified a few dominant genes governing salt tolerance in rice, and subsequent studies revealed the polygenic nature of the trait, involving both additive and non-additive gene actions [61,68,69,70,71]. Further studies found out that crop plants respond differently to salt stress in a growth stage-specific manner, making it more complex to understand the phenomenon. From such studies, it became clear that salinity stress during seedling and reproductive stages are critical to achieving better grain yield [72]. However, the salt tolerance mechanisms did not appear to be common across the identified genetic donors [73]. Therefore, understanding the mechanisms underlying the trait became a focal point of crop improvement schemes to develop salt-responsive cultivars. Development of molecular/DNA markers and software-enabled genome-wide mapping of markers combined with high throughput statistical tools paved the way for the identification of quantitative trait loci (QTLs) governing quantitate (polygenic) traits [61,74].

### Identification and Introgression of QTLs Controlling Salt Tolerance

QTL refers to any genomic region containing one or more genes that contribute to the phenotypic variation of a trait. Several molecular markers in the form of isozymes and DNA markers such as RFLP, RAPD, SSR, AFLP, VNTRs, CAPS, and RAD-Seq, have been employed in QTL mapping studies [75]. Using phenotypic parameters and marker genotyping data obtained from a suitable mapping population, genetic linkage studies are carried out to identify markers that are tightly linked to the trait under study. Different segregating mapping populations have been used for QTL mapping, including Recombinant Inbred Lines (RILs), Doubled Haploids (DH), F2 progeny, F3 progeny, back and cross-derived lines. Molecular markers have been used to generate marker linkage maps depicting the presence of each marker on the respective chromosome (also called a linkage group), and this information has been used to identify marker-trait linkages. One major advantage of QTL mapping is that marker genotypic data for mapping populations like RILs or DH need to be generated only once. The same data can be used to map QTLs for multiple traits as long as the parents used to generate the mapping population differ for those traits.

Salinity tolerance is a polygenic trait and is also significantly influenced by the environment. QTLs for salinity have been mapped in different crop species like rice [76], wheat [77], barley [78], tomato [79], chickpea [80], and soybean [81]. Through QTL mapping, multiple genes/loci associated with salinity tolerance in rice and their chromosomal locations have been identified, and these findings helped in the improvement of the trait [76]. These studies used several landraces that are tolerant to salt stress, including Pokkali, a well-studied salt-tolerant donor. Some studies identified QTLs (e.g., *Saltol* QTL) associated with seedling stage salinity tolerance [57], while others identified QTLs associated with reproductive stage salinity tolerance; for example, Hossain et al. [82] identified 16 QTLs associated with salinity tolerance during the reproductive stage using mapping population derived from cultivars Cheriviruppu and Pusa Basmati 1. The identified loci are distributed among chromosomes 1, 7, 8, and 10, and they contributed to salinity tolerance through alterations in Na^+^ uptake, pollen fertility, and Na^+^/K^+^ ratio. Similarly, more QTLs were identified that are associated with tolerance to salt stress during reproductive stages [83,84,85].

In wheat, Diaz De Leon et al. [86] identified 36 QTLs associated with salinity stress, with 13 of them being major QTLs. Of these, eight major QTLs were reproducible across two seasons under salinity stress. Likewise, several other groups have identified various QTLs in wheat governing shoot and root traits, leaf Na^+^ exclusion, K^+^ accumulation, K^+^/Na^+^ ratio, and related traits under salinity stress using DH population or RILs [77,87,88].

Barley is one of the most tolerant crops for salt stress and is an ideal model crop plant for studies on physiological and molecular mechanisms of salt tolerance. Several QTLs for various agronomic and physiological traits were detected in Barley for salinity tolerance. A significant QTL QSl.TxNn.2H associated with salt tolerance was detected on chromosome 2H [89], and about 14 more QTLs were detected on six different chromosomes [90,91]. In these studies, salinity tolerance was found to be significantly influenced by waterlogging stress, daylight length, and temperature. Furthermore, genes regulating flowering time were found to be significantly associated with QTLs governing salinity tolerance. Similarly, several dozens of QTLs associated with salinity tolerance have been identified in chickpea, soybean, and other crop plants using intra- and interspecific mapping populations (Table S1). It will be interesting to know how the genes/loci associated with salinity tolerance are conserved across related and diverse plant species.

A major purpose of identifying QTLs is to employ marker-assisted introgression of desirable QTLs from donor parents into promising genotypes. For a successful trait enhancement, QTLs showing major and reproducible effects should be selected for introgression. Several promising QTLs have been introgressed in multiple plant species for augmenting salinity tolerance. For example, *Saltol* QTL was successfully introgressed into several elite rice varieties of different countries including India, [92,93], Vietnam [94,95,96,97], Bangladesh [98], Russia [99]. *Saltol* QTL enhanced salinity tolerance of the recipient genotypes.

Salt tolerance in wheat is governed by the key gene *Kna1* from the “D” genome associated with low shoot Na^+^ transport and high affinity of K^+^ over Na^+^ [100,101]. The *Nax1* gene on chromosome 2A [102,103] and the *Nax2* gene on chromosome 5A [104] that play a role in limiting Na^+^ concentrations in the shoot, were introgressed from *Triticum monococcum* into durum wheat; *Triticum turgidum* ssp. *durum* [105], which were further introgressed into hexaploid bread wheat (*Triticum aestivum*) via interspecific hybridization and marker-assisted selection [16].

## 4. Genomic Approaches for Enhancing Salinity Tolerance

Narrow genetic diversity among cultivated gene pools in most crops is one of the major and most evident constraints on improving crop productivity. Preferential selection for some of the selected traits has created breeding bottlenecks, which, coupled with domestication, has become a major cause for the ever-dwindling genetic diversity among several crop plant species [11,106]. This major constraint restricts breeding programs’ success, resulting in a lower genetic gain rate. Global gene banks that hold huge germplasm wealth for all the crops provide a solution to counter the issue of narrow genetic diversity [107]. Germplasm stored in gene banks serves as a source of allelic variations and superior haplotypes for key traits based on the overall genetic variation for a particular species. Cataloguing genomic variation present in an entire species will help understand the genome evolution and the genetic basis for different traits of interest. It will also aid in identifying/developing markers associated with various traits to be used in the marker-assisted selection of desirable traits. Recent advances in DNA sequencing technologies such as next-generation sequencing (NGS) [108], Nanopore sequencing [109], Single-Molecule and Real-Time (SMRT) sequencing [110] have made genome sequencing feasible for all species. The new technologies have increased the pace and length of individual sequencing reads beyond the current limit of Sanger sequencing technology. They also can accelerate genome sequence assembly, reduce sequencing cost, enable accurate sequencing analysis of repeat-rich areas of the genome, and reveal large-scale genomic complexity [110]. Using these sequencing technologies, gene banks can be exploited to bring genetic diversity into the cultivated gene pool for increasing crop productivity by harnessing genetic variation latent in the large pool of diverse landraces and wild relatives [107,111].

NGS-based genome sequencing efforts have led to the decoding of genome architecture resulting in the development of a larger set of genomic resources, which enabled dissection of the underlying mechanisms or genetic basis for functional characterization of several genes in diverse plant species [112]. NGS technologies have been deployed to generate genomic information on germplasm stored in genebanks for rice [113], barley [114], pigeonpea [115], and chickpea [111]. With the availability of sequencing-based trait mapping using a biparental population or germplasm, candidate genes for salt stress response are identified [66]. Whole-genome sequencing (WGS)-based identification of genetic variation coupled with precise phenotypic data have been used to perform genome-wide association studies (GWAS). Collectively, such analyses have played a pivotal role in establishing marker-trait associations, identification of superior alleles and haplotypes for several key traits in major crop plants, including rice [116,117], wheat [118], maize [119,120,121], chickpea [111], pigeonpea [115] and common bean [122]. It is now possible to develop improved high-yielding varieties using haplotype-based breeding [11,123]. Moreover, genetic/genomic information about superior haplotypes will help select parental lines with preferred alleles at each locus, which can then be integrated into breeding programs to custom-design crops with desired allelic combinations to develop superior purelines or hybrids [10].

NGS technologies have already been proven useful for dissecting several important traits, including salinity. Due to the complex nature of stress response, it is critical to understand the complex genetic architecture of plant response’s mechanism when exposed to salinity stress. NGS technologies-based genome-wide association (GWAS) mapping has been successfully used to identify the marker(s)/gene(s) associated with salt stress responses in rice [124,125], cotton [126], barley [127], and rapeseed [128]. In addition, SNP marker-based QTL mapping studies have also identified marker(s) associated with salt tolerance in wheat [129], maize [130], chickpea [66,131], and brassica [132]. These trait mapping studies using linkage mapping and GWAS have identified and characterized several salt-responsive potential candidate genes that can be utilized to develop improved salt-tolerant varieties. More comprehensive GWAS studies involving a larger population of plant genotypes derived from crossing multiple parents called Multi-parent Advanced Generation Inter Crosses (MAGIC) lines are proposed for in-depth identification of marker-trait associations [133,134]. In addition to conventional trait mapping, NGS technologies have also been used for transcriptomics [135,136,137,138], proteomics [139], and pre-mRNA splicing [140] analysis for the identification of genes associated with salt stress responses.

For many crop plant species, it is cheaper now to genotype a breeding line at high density than to phenotypically evaluate its performance in the field. Access to improved sequencing and low-cost genotyping technologies have lent new avenues to leverage genotypic information in breeding [11]. Genome-wide sequencing combined with precise phenotypic data has been further exploited to estimate what is called ‘genomic estimated breeding values’ (GEBVs), with the help of which plant breeders can identify superior offspring for generation advancement and potential use as donors in breeding programs. In the context of genome-wide prediction, the use of GEBVs promises to help accelerate the rate of genetic gain in breeding. Genomic selection (GS) calculates the GEBV of lines using genome-wide marker profiling and allows the selection of lines before field-phenotyping, thereby shortening the breeding cycle [141,142]. GS has been successfully deployed to develop superior varieties in a cost- and time-effective manner in major crops like maize [121,143], wheat [144,145], rice [146,147], barley [148,149,150], chickpea [151,152,153] and groundnut [154]. Furthermore, the recently-popularized speed breeding technique, which hastens plant growth and development, could be applied to reducing breeding cycle time and accelerate crop research [155,156]. Speed breeding involves rapid generation advances, which can be achieved through maintaining the specific temperature, photoperiod, humidity, and harvesting and germination of immature seeds [155,157].

## 5. Genetic Engineering for Salinity Tolerance in Plants

When plants encounter salt stress conditions, several genes with different functions are upregulated or downregulated, resulting in various developmental and physiological processes that regulate stress-associated growth and metabolic changes. The proteins encoded by some of the up- or downregulated genes play an important role in the manifestation of salt-stress sensing and signal transduction pathways followed by the expression of a wide range of downstream salt stress-responsive genes, which include those that encode ion transporters and channels, enzymes involved in osmolyte biosynthesis, antioxidant systems, protective proteins such as late embryogenesis abundant (LEA) proteins. [158]. Multiple studies have highlighted the importance of transcription factors (TF’s) belonging to TF families of ERF/AP2, bZIP, MYB, MYC, NAC, WRKY, and zinc-finger proteins as regulatory elements in modulating salt stress responses [159,160].

Recent genetic engineering approaches being deployed to understand salinity tolerance include transcriptome analysis under salt stress, modification of signaling and regulatory elements, evaluation of potential genes from different metabolic pathways conferring salt tolerance, analysis of post-transcriptional modifications, studies on epigenetic regulation, and genome editing for precise and targeted genetic engineering. Overexpression of the positive regulators and downregulation/disruption of negative regulators of salt tolerance are commonly used genetic engineering approaches adopted to study and to improve the trait. However, such genetic engineering approaches entail prior identification and understanding of the gene(s) involved in controlling the trait of interest. Manipulation of several genes associated with ion homeostasis, compatible-solute biosynthesis, and antioxidant metabolism for improving salt stress have been attempted extensively [161,162,163,164].

### Genetic Manipulation of Ion Transporters and Other Genes Associated with Salinity Tolerance

Ion transporters play an important role in salt tolerance through the regulation of transport and ion homeostasis. They deter ion toxicity by restricting the uptake and transport of harmful Na^+^ and Cl^−^ by efflux or by compartmentalizing these toxic ions into vacuoles by ion transporters, including Na^+^ antiporters (NHXs). They also help maintain beneficial K^+^ ion homeostasis in the cytoplasm through plasma membrane-bound high-affinity potassium (K^+^) transporter (HKT). However, most of the transporters have tissue- and organ-specific expression patterns in many species and depend on the developmental stage and stress levels [165].

Single and multiple ion-transporter genes obtained from various sources have been engineered to improve salt tolerance in different plant species, including Arabidopsis, rice, tobacco, cotton, and soybean, among other crops [166,167,168,169,170,171]. In *A. thaliana*, overexpression of vacuolar Na^+^/H^+^ antiporter (AtNHX1) resulted in increased salinity tolerance via enhanced vacuolar sequestration of Na^+^ into the cytoplasm avoiding the toxic accumulation of Na^+^. Similarly, overexpression of AtNHX1 and related NHX proteins imparted enhanced salt tolerance in brassica, wheat, cotton, tobacco, tomato, and soybean [167,172].

In addition to Na^+^ and K^+^ transporters, proton pumps constituting plasma membrane proton (H^+^)-ATPases, vacuolar membrane H^+^-ATPases, plasma membrane, and vacuolar membrane H^+^- pyrophosphatases (H^+^-PPases) also play an important role during salt stress tolerance. Understandably, halophytes have been exploited as a major source of genes to impart salt tolerance in several plant species. For example, expression of a vacuolar H^+^-ATPase subunit c1 (SaVHAc1) gene from the halophyte grass *Spartina alterniflora* led to increased salt stress tolerance in rice [173]. The observed salinity tolerance was due to the sequestration of Na^+^ ions at the tonoplast by Na^+^–H^+^-antiporter that was energized by a proton motive force, aided by the overexpression of SaVHAc1 gene. The tolerance was also accompanied by the maintenance of net photosynthesis with higher growth rates and grain yield under salt stress. These proton pumps produce electrochemical potential gradients essential for root nutrient uptake and cell growth [174]. In another study, lower oxidative stress due to improved ion homeostasis in Arabidopsis overexpressing PM H^+^-ATPase (SpAHA1) of the halophyte *Sesuvium portulacastrum* conferred salt tolerance as measured by enhanced seed germination ratio, root growth, and biomass [175]. The levels of salt tolerance were even higher when SpAHA1 was co-expressed with *SOS1* [176]. In the cyanide-resistant respiration pathway in plant mitochondria, Alternative oxidases (AOXs) are the terminal oxidases that play a vital role in abiotic stress and are proposed as a functional marker for high stress-tolerant breeding. Overexpression of AOX in rapeseed (*BnaAOX1b*) enhanced tolerance to osmotic and salt stress [177].

Other than the metabolic genes, researchers have also attempted to exploit master switches such as regulatory elements and signaling molecules to enhance salt stress tolerance. However, the engineering of these molecules often results in pleiotropic effects because of their involvement in multiple pathways governing plants’ growth and metabolism. On another front, the expression of various transcription factors (TFs) has been modulated to impart salt stress tolerance in plants [159,178]. For example, constitutive expression of the transcription factor SALT-RESPONSIVE ERF1 (*SERF1*) improved salt stress tolerance in rice by regulating the expression of other regulatory genes such as *MAP3K6*, *MAPK5*, *DREB2A*, and *ZFP179* [179]. By and large, these genes are involved in various stress responses, and their underlying mechanisms are fairly well understood. Several overexpression studies in multiple plants species such as Arabidopsis, rice, and soybean demonstrated the involvement of other TFs such as *OsAP21*, *SbAP37*, *GmDREB6*, and *OsMYB6* in salt stress tolerance, and in some cases, it was accompanied by accumulation of proline [180,181,182,183].

Several genes used for genetic engineering of salt tolerance across different plant species are listed in Table S2. However, notwithstanding various efforts, the desirable success obtained under controlled conditions could not be replicated in field experiments to a similar degree because of the multigenic nature of the trait and the complexity of stress. Unlike other abiotic stressors, salt stress is persistently present entailing plants to modulate a different set of metabolic pathways and the underlying genetic machinery in a tissue-specific and growth stage-specific manner. The scenarios could become more complex if plants undergo other abiotic/biotic stresses simultaneously. Therefore, understanding gene regulatory networks and epigenetic regulatory mechanisms underlying salt stress can pave the way for improving the trait across plant species.

## 6. Genome Editing to Enhance Salt Tolerance in Plants

Genome editing (GE) approaches are among the recently-developed genetic engineering tools that allow us to modify one to few base pairs of a specific gene/locus (to create knockout or knockdown mutants), substitute an antecedent allele with another orthologous allele originating from a related species (for allele correction), and facilitate the introduction of foreign genes into pre-defined genomic regions (to create knock-in mutants) [184]. However, prior identification of potential positive and negative regulators of the trait of interest is essential for the specific targeting of genes by GE tools [12]. Typically, findings from transcriptome analyses (e.g., RNA seq) of plants subjected to specific conditions and genome sequence analysis are being used to identify potential regulators of a given trait as targets for genome editing.

**Figure 1:**
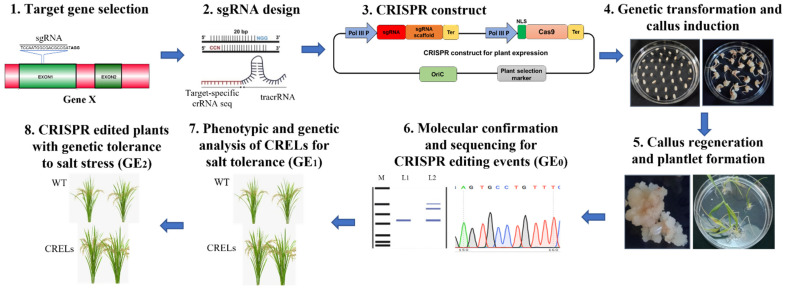
A scheme of CRISPR/Cas mediated genome editing for salt tolerance in plants. CRELs: CRISPR Edited Lines. NLS: Nuclear Localization Signal (NLS can also be at the end of Cas9), OriC: Origin of Replication C, Ter: Terminator, Pol III P: Polymerase III promoter, GE0: Genome Edited Generation 0, GE1: Genome Edited Generation 1, GE2: Genome Edited Generation 2.

Site-specific endonuclease-based mechanisms, such as transcription activator-like effector nucleases (TALENs), zinc finger nucleases (ZFNs), and clustered regularly interspaced short palindromic repeats (CRISPR)/CRISPR-associated protein 9 (Cas9), are the most widely used GE approaches [185]. Evidently, CRISPR/Cas method has become the most preferred GE tool because it is relatively inexpensive, quicker, precise, and enables several locations across the genome to be edited concurrently [186]. Since the CRISPR-Cas9 has been established for use in model and crop plant species, consistent paradigm shifts have permitted the generation of transgene-free edited plants [187]. The CRISPR/Cas in plant genome editing has been mainly used to generate indel mutations via error-prone nonhomologous end joining (NHEJ) repair of DNA double-strand breaks (DSBs) to create loss-of-function (knockout) or reduced-function (knockdown) mutants. The mutants developed through GE can be construed as transgene-free since the genome-edited lines are expected to be devoid of CRISPR-Cas-associated transgenes. Therefore, genome-edited plants may suffer less from regulatory concerns compared to transgenic plants. CRISPR/Cas method has been widely employed to understand genetic and molecular mechanisms of abiotic stress tolerance in plants and, in some cases, to improve the trait. A diagrammatic representation of various steps of CRISPR/Cas9-mediated genome editing for developing salt tolerance in plants is shown in Figure 1.

Several putative negative regulators of the salt stress response, previously identified from other studies, have been targeted by the CRISPR system to increase the salt stress tolerance of plants. Some recent examples include the targeting of *OsRR22* and the SQUAMOSA promoter-binding like protein 10 (*OsSPL10*) in rice [188,189]. *OsRR22* encodes the type B response regulator transcription factor, which participates in cytokinin-mediated signal transduction and metabolism. A previous study had also reported a significant enhancement of salt stress tolerance in *Osrr22* loss-of-function mutants of rice [190]. For similar purposes, auxin response factor 4 (*SlARF4*) and Hybrid proline protein (SlHyPRP1) were targeted in tomato [191,192,193]. Knockout of auxin response factor 4 (*SlARF4*) in tomato enhanced osmotic and salinity stress tolerance through reduced stomatal conductance increased leaf water content, and ABA production [191]. Notably, Tran et al. [193] employed a multiplexed CRISPR-Cas9 system with multiple guide RNAs (gRNAs) to precisely eliminate functional domains of SlHy-PRP1 to enhance salt stress tolerance. SlHyPRP1 expresses differentially in response to various stress signaling molecules such as H_2_O_2_, NO, and phytohormones, suggesting their involvement directly or indirectly in different defense-responsive signaling pathways in tomato. A list of genes targeted by CRISPR/Cas9 system resulting in enhanced salinity stress tolerance in major crop plants is presented in Table 1.

**Table 1:** List of some of the genes targeted by the CRISPR method of genome editing for the genetic enhancement of salt tolerance in major crop plants.

Crop Plant Species	Target Genes	Gene Function	References
Arabidopsis (*Arabidopsis thaliana*)	*AITR*	ABA-induced transcriptional repressor	[194]
	*CBF*	C-repeat binding factor	[195]
	*SIZ1*	C2H2 type zinc finger protein	[196]
Tomato (*Solanum lycopersicum*)	*SP5G*, *SP*	Day length sensitivity regulators	[197,198]
	*WUS*	Act as both transcriptional activator and repressor of genes in the shoot apical meristem	[197]
	*GGP1*	Vitamin C synthesis	[197]
	*HKT1;2*	High affinity potassium transporter	[199,200,201]
	*ARF4*	Auxin signaling	[191]
	*HyPRP1*	Multistress tolerance	[192,193]
	*CLV3*	Regulates shoot and floral meristem development	[197,202]
Maize (*Zea mays*)	*HKT1*	High affinity potassium transporter	[203]
Rice (*Oryza sativa*)	*DOF15*	Transcription factor	[204]
	*NCA1a*, *NCA1b*	Catalase activity-regulating chaperone	[205]
	*PQT3*	Ubiquitin ligase	[206]
	*FLN2*	Involved in sucrose metabolism	[207]
	*BBS1*	Chaperone-mediated signaling	[208]
	*NAC041*	Transcription factor	[209]
	*BG3*	Cytokinin transporter	[210]
	*MIR528*	Salt stress response regulator	[211]
	*DST*	Zinc finger transcription factor	[212]
	*SPL10*	Transcription factor	[188]
	*RR9, RR10*	Cytokinin signaling	[213]
	*RR22*	Transcription factor	[189,190]
	*OTS1*	Salt stress response regulator	[189,214]
	*SAPK1, SAPK2*	ABA signaling regulator	[215]
	*PIL14*	Transcription factor	[216]
Soybean (*Glycine max*)	*MYB118*	Transcription factor	[217]
	*NAC06*	Transcription factor	[218]

Additionally, CRISPR-based technology can be deployed to generate elite allelic variants for the genes that play a key role in salt stress tolerance.

Although homology-directed repair (HDR)-mediated knockin or allele replacement of positive regulators of salt stress response to enhance tolerance towards salt stress is a viable option, very few examples of such studies are available. This is probably because CRISPR-mediated knockin experiments are more challenging than the simple binary vector-mediated introduction of genes into plant genomes. Recently, Vu et al. [199] demonstrated successful execution of the HDR strategy for allele replacement of high-affinity K^+^ transporter 1;2 (HKT1;2) in tomato using the CRIPSR/Cpf1 system. HKT1;2 plays a key role in maintaining K^+^ uptake under salt stress [200]. According to a previous study in tomato, N/D variant (N217D) in the pore region of HKT1;2 determines salinity tolerance [201]. This pore region of HKT1;2 determines selectivity for Na^+^ and K^+^. Vu et al. [199] generated tomato line that carried the salt-tolerant allele (N217D) of HKT1;2 using CRIPSR/Cpf1-mediated HDR strategy involving geminivirus-based replicons as carriers of the donor allele. These plants showed improved tolerance when exposed to salt stress (up to 100 mM NaCl) [199]. Although this study involved the generation of plants that are transgenic for CRISPR-Cas constructs, allele replacement (knock-in) can also be accomplished using transgene-free editing, as discussed below in Section 6.1.

CRISPR-based approaches can also be employed to create knockout mutations for functional characterization of genes that play an important role in salt stress tolerance. The genes which are putative negative regulators of the salt stress response are either downregulated or knocked out using the CRISPR-Cas approach to investigate their mechanistic role in responses to salt stress. RNAi approaches have also been used to downregulate putative negative regulators of response to salinity stress. For example, RNAi knockdown of transcription factor *SlMBP8* significantly improved salinity and drought stress tolerance in tomato [219]. Promising candidate genes from RNAi knockdown studies can serve as excellent targets for future CRISPR-Cas-based editing.

### 6.1. Current Challenges and Opportunities with CRISPR-Based Approaches

While designing gRNA sequences, care should be taken to avoid off-target or unintentional mutations. However, off-target mutations that occurred during genome editing can be detected by targeted deep sequencing [220]. Moreover, the off-target sites can also be predicted based on similarity to the gRNA using bioinformatics software such as CRISPR-P, Cas-OFFinder, Benchling, CGAT, and CRISPR-P 2.0 [221,222]. Modified Cas proteins such as Dead cas9 (dcas9) [223], SpCas9n (Cas9n) [224], and FokI Cas9 (fCas9) [225] have also been used to reduce the off-target mutations. Also, Cas9 proteins with enhanced non-target cleavage capability are being isolated from various bacterial strains of novel- and stretched-PAM sequences. Inadequate optimization of Cas9 codons may also generate off-target mutations, but this can be minimized by using a codon-optimization based on codon usage in plants [226,227].

Lately, the CRISPR/Cpf1 system from *Francisella novicida* has drawn traction as a preferable alternative GE tool to CRISPR/Cas9 [228]. Cpf1 (also known as Cas12a) is a smaller endonuclease than Cas9, and it takes shorter CRISPR RNA (crRNA) to function effectively [229]. Cpf1 is a single-stranded RNA-guided effector nuclease protein that binds upstream of the protospacer adjacent motif (PAM) and introduces 5 base pair (bp) staggered cuts into the DNA at the proximal end of the PAM, far away from the seed region. During the conversion of Cpf1-associated CRISPR repeats to mature crRNAs, the CRISPR/Cpf1 model does not require trans-activating crRNA (tracrRNA) [230]. This system successfully cleaves target DNA conveniently close to a short T-rich PAM, whereas Cas9 only works with a G-rich PAM.

Very recently, a hyper-compact genome editor called CRISPR/CasΦ has been discovered that uses a single active site for both crRNA processing and crRNA-guided DNA cleavage for targeting foreign nucleic acids. This strategy is effective in in vitro, animal, and plant systems with greater target recognition potential than other CRISPR/Cas proteins. Furthermore, the molecular weight of CasΦ protein is approximately half of the Cas9 and Cas12a, which makes it convenient for delivery into the host organism [231]. Therefore, CRISPR/CasΦ system can serve as an effective alternative to CRISPR/Cas system for future GE requirements.

The majority of plant traits are governed by multiple interacting genes, and several genes exist as gene families with their members possessing overlapping functions. Therefore, modification of a single gene belonging to a gene family does not necessarily result in a desirable phenotype. As a solution to this problem, multiplexed GE orchestrated by CRISPR/Cas9 [232] was designed that allows multiple sgRNA cassettes to be designed into a common vector framework driven by single or multiple promoters. This approach enables the editing of multigenic agronomic traits as well as simultaneous editing of a gene family in plants.

CRISPR-based HDR strategy is more challenging compared to the one that involves NHEJ-based genome editing because the success of HDR not only depends on the precise cleavage of the target sites but also on the precise homologous recombination between the target site and the donor DNA. Moreover, most of the reported cases of geminivirus replicon-based HDR strategy have entailed selection markers associated with the edited alleles, which is still challenging [233,234,235]. Further, the effective application of replicon cargos in editing plant genomes has been demonstrated to be limited by their size [236,237]. Recent advances in CRISPR technology are aimed at addressing some of these challenges.

Several methodologies have been developed to easily isolate transgene-free edited plants. He et al. [238] employed an interesting strategy in which BARNASE and CMS2 genes were used as suicidal genes to eliminate embryos and pollens containing the transgene in T_0_ plants. BARNASE encodes a toxic protein with a nuclease activity while CMS2 (also called ORFH79) disrupts mitochondrial functions during male gametophyte development resulting in male sterility [238]. One more method used to obtain transgene-free edited plants makes use of a transient expression of CRISPR/Cas9 DNA or RNA, as demonstrated by Zhang et al. [239] in wheat. In this method, identification of gene-edited mutants does not require selectable markers, and homozygous edited mutants can be obtained in T_0_ generation without the incorporation of exogenous DNA into the plant genome. Currently, more approaches are available to generate transgene-free genome-edited plants [240,241,242].

A Tobacco Mosaic Virus RNA (TRBO) has recently been used to transiently overexpress guide RNA in *Nicotiana benthamiana* to enhance the genome editing efficiency of the CRISPR-Cas system [243,244]. This vector can be employed in all of the above-discussed genome editing strategies to improve efficiency. We are currently testing this strategy to increase genome editing efficiency in rice and tomato.

## 7. Epigenetic/Epigenomic Approaches to Enhance Salinity Tolerance

Improvement of various agronomic traits, including salt tolerance of crop plants, had over the decades been accomplished mainly by utilizing genetic variation that existed among crop plants, often tapping into genetic variation latent among wild varieties/species of crop plants. However, the loss of several natural habitats of crop plants combined with consistent usage of natural genetic resources has persistently dwindled the sources of genetic variation [245,246]. Lack of genetic variation for various crop plants has currently become a major limiting factor in plant breeding endeavors. To address this problem, a battery of chemical mutagens and radiation treatments have been used to create genetic variation. The mutations induced through such approaches ranged from point mutations to large-scale chromosomal aberrations. Notwithstanding efforts to create genetic variation, the creation of desirable alleles has not been possible for several traits at the desired pace. Rapid generation of genetic variation appears to be a significant requirement to tackle the fast-changing climatic and soil conditions. Traditional methods of crop improvement are not only time-consuming but also suffer from additional bottlenecks. First, it is extremely challenging to separate desirable alleles from undesirable alleles, which are tightly genetically linked. Second, some chromosomal regions act as suppressors of crossing over, further strengthening the linkage among multiple alleles/loci. Current strategies include screening a large number of individuals to identify desirable segregants, but this approach is not feasible in many cases due to its time- and labor-intensive nature. To address these challenges, we propose and discuss novel strategies based on recent advances in the field of epigenetics/epigenomics to create heritable epigenetic variation in crop plants for the improvement of traits of significance, including salt tolerance [247]. Note that several studies have reported the involvement of modulated DNA methylation and histone modifications in salt stress tolerance [248], reviewed in [249], and therefore, the epigenetic variants we describe below are expected to serve as valuable tools in improving tolerance to salt stress.

### 7.1. Development of Epigenetic Recombinant Inbred Lines (EpiRILs)

**Figure 2:**
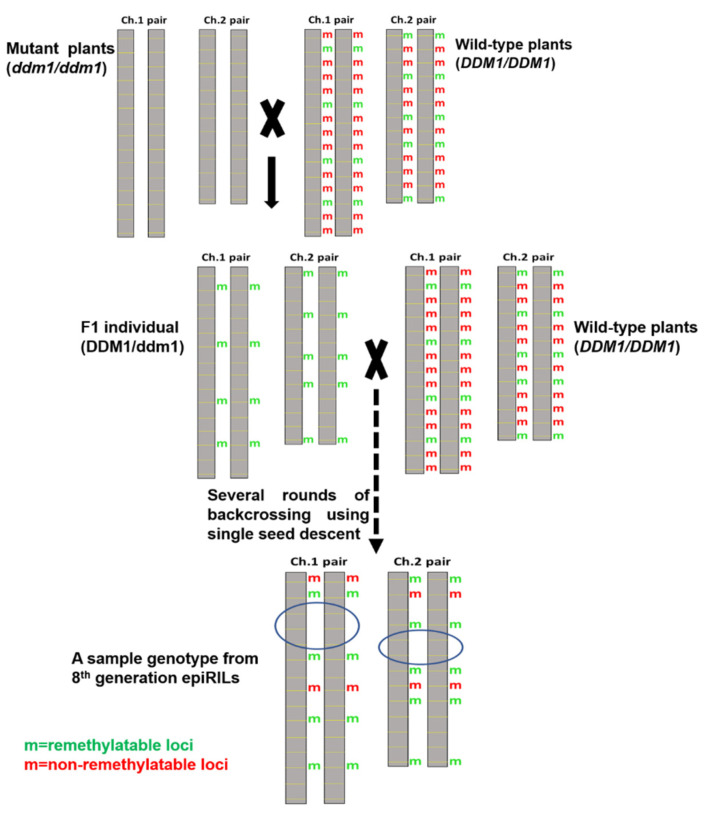
A flowchart illustrating a scheme for the development of epigenetic recombinant inbred lines (epiRILs). Circled loci are examples of loci that do not regain methylation after the introduction of the wild-type DDM1 gene, resulting in the formation of new epialleles.

Epigenetic Recombinant Inbred Lines (epiRILs) are similar to traditional recombinant inbred lines (RILs), but epiRILs vary for differentially methylated regions (DMRs) while RILs carry allelic variations. For other differences between epiRILs and RILs, see Table 2. EpiRILs were first created in the model plant *Arabidopsis thaliana*, and these epiRILs showed heritable epigenetic variation for several traits of agronomic importance [250]. A diagram depicting the scheme for the generation of epiRILs and the principle underlying the creation of epigenetic variation in epiRILs is given in Figure 2.

**Table 2:** Comparison between Recombinant Inbred Lines (RILs) and Epigenetic Recombinant Inbred Lines (epiRILs).

Recombinant Inbred Lines (RILs)	Epigenetic Recombinant Inbred Lines (epiRILs)	Related References Pertaining to epiRILs
1. Mainly vary genetically; each RIL has a different combination of alleles.	1. Mainly vary for epialleles (variation with respect to epigenetic marks like methylation, acetylation, and others. Each epiRIL has a different combination of epialleles	[250]
2. QTLs governing a trait can be identified and introgressed into a genotype of choice	2. epiQTLs governing a trait can be identified and introgressed into a genotype of choice	[251]
3. Typically, the parents involved in the generation of RILs are genetically diverse	3. The parents involved in the generation of epiRILs can be isogenic or near-isogenic, or genetically diverse, but they differ significantly for the epigenome	[250]
4. No need to create/induce specific mutations in parents to create RILs	4. To create epiRILs, one of the parents should be an epigenetic mutant	[250]
5. In RILs, genetic variation can also bring in some epigenetic variation, particularly when the variation is related to an epigenetic modifier. However, such a variation has not been systematically documented in RILs.	5. In epiRILs, epigenetic variation can also cause genetic variation by enhancing meiotic crossing over and activation of transposons	[250,252]
6. Most of the genetic variation of RILs is heritable	6. In epiRILs, some epigenetic variation is heritable (not all)	[250,253,254,255,256]

For creating epiRILs, wild-type (WT) *A. thaliana* (ecotype; Columbia-0, also called Col-0) plants were crossed with mutant arabidopsis plants, which carried knock-out mutation for DDM1 (Decrease in DNA Methylation 1) gene, which is a chromatin remodeler known to affect DNA methylation in all the three cytosine contexts (CG, CHG, CHH). DDM1 is mainly involved in the maintenance of DNA methylation and the silencing of repeat elements, including transposons [257,258,259,260]. A single F_1_ plant obtained from Col-0 WT (*DDM1/DDM1*) x *ddm1* mutant (*ddm1/ddm1*) cross was backcrossed as a female parent to the Col-0 WT line. From the resulting progeny, 500 individual plants with DDM1/DDM1 genotype were chosen to generate about 500 epiRILs through six rounds of propagation through the single seed descent method with no selection bias.

It is important to note here that when the WT DDM1 gene was introduced into *ddm1*/*ddm1* plants, DNA methylation was not restored at all the loci where methylation was lost due to loss of DDM1 function. This lack of restoration of DNA methylation at some genomic loci formed the basis for the generation of epialleles across the genome. In these epiRILs, numerous parental DNA methylation variants, also called Differentially Methylated Regions (DMRs), are inherited for 16 generations, and the heritability studies are still ongoing [250,256]. Most importantly, these epiRILs displayed heritable phenotypic diversity for various traits, including flowering time, plant height, plant responses to defense hormones, plants’ plasticities to drought, and nutrients [250,253,254,255]. In these epiRILs, epigenetic quantitative trait loci (epiQTLs) imparting enhanced defense responses have recently been identified [251], creating a scope for introgression of such epiQTLs into other genotypes/plant lines for trait improvement. Moreover, the epigenetic diversity of these epiRILs had been attributed to increased productivity and stability of plant populations in studies where growth and productivity were compared between epigenetically uniform and epigenetically diverse populations upon challenging them with weeds and pathogens [256]. Furthermore, when used as parents for crossing, some of the epiRILs triggered heterosis in the resulting novel epigenetic hybrids (epihybrids), independent of genetic changes between the parents [261]. A recent large-scale study comprising high-quality single-base resolution methylomes and transcriptomes from 1001 Genomes collection of *A. thaliana* revealed that geographic origin is a major predictor of genome-wide DNA methylation and gene expression patterns caused by epialleles [262]. Given the largely conserved epigenetic mechanisms across plant species, similar geographical origin-derived epigenetic diversity is likely to occur in crop plant species as well, a factor that needs to be considered while generating epiRILs. For similar reasons, MAGIC lines (described above) can also be explored as potential parents for the development of epiRILs to generate additional epigenetic variation via the creation of epialleles from genotypes containing “mosaic genomes” of geographically diverse accessions/ecotypes [134].

Furthermore, the epiRILs showed enhanced genetic variation due to increased meiotic recombination (crossovers) [252]. Others have also observed altered crossover frequency due to loss of DNA methylation or histone 2 A.Z (H2A.Z) deposition [252,263,264,265,266]. The outcomes from these studies highlight the importance of epigenetic mutations in enhancing genetic variation by altering meiotic recombination rates, prompting approaches to use controlled recombination for plant breeding [267]. Collectively, these findings indicate significantly that every plant genotype has the potential to be improved by just manipulating its epigenomic landscape. The scope for such a plant improvement tends to be even higher for several crop plants given their larger genome sizes and larger gene families compared to the model plant Arabidopsis.

There are several DNA methyltransferases, histone modifiers, and chromatin remodelers in plants that have some overlapping functions. Some epigenetic modifications are linked; for example, histone deacetylation and histone 3 lysine 9 (H3K9) methylation are linked to DNA methylation and collectively play a major role in gene silencing [268,269]. Because most of the epigenetic modifications regulate only a subset of loci/genomic regions, the creation of epiRILs using mutations of other epigenetic modifiers will potentially generate more epialleles across the genome. Likewise, the additional epigenetic mutations will likely provide more options to modulate crossovers across the genomic regions. Currently, our research group has collaboratively embarked on generating epiRILs in rice and tomato to be utilized for the improvement of various traits, including salt stress tolerance.

### 7.2. Generation of Epigenetic Variants Using Inhibitors of Epigenetic Modifiers

Generation of epiRILs is a tedious and time-consuming endeavor but once generated, they have numerous benefits, as discussed above. We propose other non-transgenic approaches to create epigenetic variants which relatively require a shorter time. Transient inhibition of epigenetic modifiers through the application of chemical inhibitors is expected to have a similar effect compared to that of genetic mutations. Brief exposure of plant seedlings to chemical inhibitors of epigenetic modifiers will result in loss of epigenetic marks in the newly-divided cells. However, upon removing the chemical inhibitors, the lost epigenetic marks will not be likely re-established at all the genomic loci, resulting in the formation of epialleles. These epialleles can be exploited by crossing the inhibitor-treated plants showing considerable phenotypic variation with the untreated plants enabling identification of promising segregants (variants). Growing evidence suggests that phenotypes caused by epigenetic mutations are stochastic in nature [270,271], which has the potential to further enhance epigenetic variation. Different classes of inhibitors are available for various enzymes involved in epigenetic modifications, such as DNA methyltransferases, histone deacetylases, and histone methyltransferases. For example, in eukaryotes, histone acetylation and deacetylation play a vital role in gene regulation. Levels of histone acetylation are modulated by histone acetyl transferases and histone deacetylases (HDACs) [272]. HDACs have been shown to play a role in plants’ response to various abiotic stresses [272,273,274]. Several known inhibitors of histone deacetylases are commercially available, which function both in plants and animals, although each compound appears to effectively target only a subset of HDACs [275,276]. Our group has again taken a collaborative initiative to establish this non-transgenic approach for generating heritable epigenetic variation.

## 8. Role of Plant Growth-Promoting Rhizobacteria in Enhancing Salt Tolerance of Plants

The soil is a complex, heterogeneous mixture of minerals, organic matter, air, water, and live organisms, including a rich and diverse microbiome. The soil microbiota associates closely with the plant roots either by endocolonizing them or existing in the rhizosphere. These endospheric and rhizospheric microbiota, along with their phyllospheric counterparts, are part of the plant holobiont [277]. The rhizospheric microbiome comprises pathogenic as well as beneficial microbes (bacteria and fungi). These microbes have evolved to co-exist with planta by interacting with them through the exchange of chemical signals. The beneficial soil microbes restrict the growth of pathogenic microbes in the vicinity of plant roots, thus providing “bio-control” to the plants while also offering other benefits to them. These beneficial microbes, collectively termed as plant growth-promoting microbes (PGPM), include rhizobacteria (PGPR) and fungi (PGPF).

Examples of PGPR include several species of Bacillus, Pseudomonas, Rhizobium, Azospirillum, and others. They help plants in myriad ways, including growth promotion (as the name suggests) and stress alleviation (both biotic and abiotic). Research on the beneficial effects of PGPR has gained substantial momentum over the last decade. Among many abiotic stresses that PGPR are known to modulate in plants, salinity stress is one among them [278,279,280]. Based on recent literature demonstrating the involvement of several epigenetic regulatory mechanisms in plants’ responses to diverse abiotic stresses [249,281], we speculate that PGPR possibly enhances salinity tolerance not only through genetic but also by epigenetic mechanisms. We could not find any report in this regard but anticipate that future studies are likely to discover PGPR-mediated epigenetic mechanisms underlying salt stress responses in plants. Several strains of PGPR have been shown to enhance salinity tolerance in several plant species, including crop plants. For example, halotolerant PGPR strains AP6 (*Bacillus licheniformis*) and PB5 (*Pseudomonas plecoglossicida*) improved salt tolerance in sunflower by stimulating antioxidant enzymes activity [282]. Few other examples include enhancement of salinity tolerance in okra, capsicum, oat, and Arabidopsis by diverse species and strains of PGPR [282,283,284,285,286]. For a detailed review on this topic, refer to Ha-tran et al. [287]. Much like other abiotic stresses, mechanisms of salinity stress alleviation in plants by PGPR include (but are not limited to) the following broad categories.

### 8.1. Expression of Key Stress-Inducible Genes

In the past decade, certain aspects of the molecular biology of PGPR-mediated salinity tolerance in plants have been elucidated. A genome-wide expression profiling of *A*. *thaliana* inoculated with *Pseudomonas putida* (MTCC5279) helped identify a wide variety of *A. thaliana* genes regulated by PGPR, including auxin-responsive genes responsible for increased auxin production and calcium-dependent protein kinase genes involved in salt response signaling [288]. The upregulation of these genes by PGPR modulates plant growth during high salt stress conditions. In another study, inoculation of the PGPR *Arthrobacter nitroguajacolicus* in wheat has been shown *to* upregulate the expression of P450 genes, ascorbate peroxidase genes, oligopeptide transporters, ATP binding cassette, and ion transporters, collectively improving their ability to uptake nutrients and tolerate salt stress [289]. In a similar study, *Pseudomonas putida* NBRIRA inoculation in chickpea exhibited improved drought and salt stress tolerance by modulating the expression of miRNAs and their target genes [290]. Their analysis revealed the altered expression of nine conserved miRNAs in chickpea caused by PGPR inoculation under salt stress. While some were downregulated (e.g., miR169 and miR396), others were induced (e.g., miR159) at various time points post treatments. They also found altered expression patterns of the genes targeted by these miRNAs, such as MYB, TCP, and the ARF family of TFs.

### 8.2. Modulation of Stress-Induced Compatible Solutes, Phytohormone Homeostasis, and Redox Status of Plants

As discussed above, it is well known that compatible osmolytes such as proline, sorbitol, and glycine betaine, positively modulate tolerance to dehydration stress in plants, which can be induced by drought and salinity. The role of PGPR in enhancing the concentrations of such compatible solutes has been studied both under osmotic stress in general [291,292] and salinity stress in particular [284,293]. These groups observed PGPR-mediated accumulation of proline, an important compatible osmolyte, in *A. thaliana* and transgenic *Sorghum bicolor*. Since phytohormones are key components of signal transduction processes involved in abiotic stress tolerance, their roles have been implicated in PGPR-mediated salinity tolerance as well. For example, augmented growth of cucumber plants accompanied by increased cellular levels of gibberellic acid was observed both under salinity and drought conditions when inoculated with PGPR *Burkholdera cepacia SE4*, *Promicromonospora spp. SE188* and *Acinetobacter calcoaceticus SE370* [294]. One of the most important stress-modulating phytohormones is ethylene. Several PGPR strains are known to secrete the enzyme ACC deaminase, which degrades ACC released by plants, thus limiting its supply for ethylene biosynthesis. Jalili et al. [295] and Bal et al. [296] have reported the isolation of ACC deaminase producing novel PGPR strains and their ability to impart salinity tolerance in canola (*Brassica napus* L.) and rice, respectively. One of the central mechanisms of manifestation of a variety of stresses in plants is oxidative stress. It is well understood that oxidative stress due to the generation of copious amounts of ROS is a common repercussion of both biotic and abiotic stresses in plants. PGPR is able to modulate the ROS production and the scavenging pathways in plants under abiotic stress [292]. Likewise, enhancement of salinity tolerance through modulation of ROS-scavenging enzymes was observed in lettuce and oat when inoculated with PGPR *Pseudomonas mendocina* and *Klebsiella* sp., respectively [284,297].

### 8.3. Release of Volatile Compounds

Certain PGPR strains are known to release volatile compounds that aid in plant stress tolerance. For example, the PGPR strain *Bacillus subtilis* (recently renamed as *Bacillus amyloliquefaciens*) GB03 produces an array of volatile compounds [298]. This bacterial strain imparts salt tolerance to the medicinal plant *Codonopsis pilosula* by positively modulating stomatal conductance and photosynthetic rate [299]. In a similar study, bacterial volatiles have been shown to aid salt stress tolerance by modulating the function of AtHKT1 sodium transporter and reducing the levels of sodium in the whole plant [300].

The literature summarized above underscores a key role for PGPR in enhancing salinity stress tolerance in plants. However, more investigations need to be carried out to understand molecular mechanisms of signaling underlying beneficial interactions between plants and PGPR. Such mechanistic insights can potentially help in identifying additional molecular, genetic, and epigenetic components involved in salinity stress tolerance of plants which can be targeted during future genetic engineering and molecular breeding programs for further enhancement of the trait. In addition, the application of appropriate wild-type or recombinant PGPR containing ideal genomic composition to the soil can also be developed as an effective strategy to combat salt stress.

## 9. Conclusions

**Figure 3:**
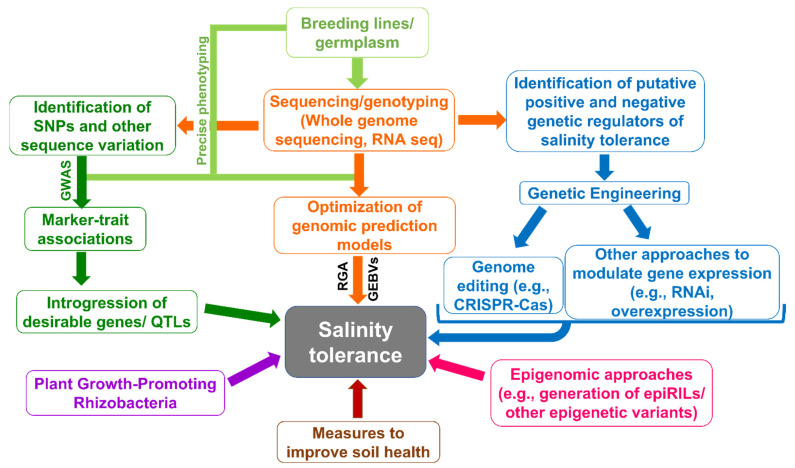
Integration of approaches to enhance salinity tolerance in plants. EpiRILs, Epigenetic recombinant inbred lies; GEBV, Genomic estimated breeding value; GWAS, genome-wide association studies; NGS, Next generation sequencing; QTL, Quantitative trait locus; RGA, Rapid generation advance; RNAi, RNA interference SMRT, Single molecule real-time sequencing; SNP, Single nucleotide polymorphism.

Globally, soil salinity is one of the formidable challenges plants are facing, resulting in significant plant yield losses and reduced soil health. More than 50 years of research on salinity has resulted in a fair understanding of the multifarious aspects of the salt stress biology of plants, including causes, consequences, and mechanisms of stress tolerance at the molecular level. In this comprehensive review, we provided a panoramic view of diverse approaches employed to bolster plants’ ability to tolerate salt stress, including some new approaches and futuristic perspectives (Figure 3). Moving forward, integrating multiple approaches as discussed in this review could provide more effective and long-lasting solutions in tackling the problem of soil salinity. Lastly, we think that it is equally important for the international community to take measures to improve and maintain soil health to minimize the detrimental effects engendered by soil salinity.

## Data Availability

Not applicable.

## Supplementary Materials

The following are available online at https://www.mdpi.com/article/10.3390/biology10121255/s1, Table S1: List of QTLs identified that were associated with salinity tolerance. Table S2: List of genes manipulated to enhance salinity tolerance. Supplemental references: Additional literature referred to in the supplemental tables.
