# Supplementary material for: Genetic, Epigenetic, Genomic and Microbial Approaches to Enhance Salt Tolerance of Plants: A Comprehensive Review
Source: Biology (Basel). 2021 Dec 1;10(12):1255. doi: 10.3390/biology10121255 (PMC8698797; doi:10.3390/biology10121255)
Supplement: Supplementary file 1 [file biology-10-01255-s001.zip › biology-1463012-supplementary.pdf]

Table S1. List of QTLs associated with salinity tolerance that were identified in different crop plants.

BC, Backcross; BILs, Backcross inbred lines; DH, Doubled haploids; F, Filial; IL, Isogenic lines; NILs, Near isogenic lines RILs, Recombinant Inbred lines.

S.No	Parents used for crossing	Mapping Population used	Size of the mapping population	LOD score for the QTLs	QTL contribution to phenotypic variation (%)	No.of QTLS detected	References
**Rice**							
1	Fixed line	Fixed line	1			1	Claes et al.(1990)
2	IR 29/ Pokkali	F_8 RILs	80	>3- 17.3	5.86- 80.2	11	Gregoria (1997)
3	Tesanai 2/CB	F_8 RILs	108	3	5.8-17.45	18	Lang et al. (2001B)
4	IR55178 (IR4630-22-2-5-1-3/IR15324-117-3-2-2)	F_6 RILs	118	3	6.4-19.6	11	Koyama et al. (2001)
5	IR 29/ Pokkali	F_8 RILs	54	5.8-6.8	39.2-43.9	3	Bonilla et al. (2002)
6	IR 29/ Pokkali	BC_3F_4 NILs	3000	3.34-5.02	0.44	2	Niones (2004)
7	Nona Bokra/Koshihikari	F_2:F_3	133	3.32-11.74	12.4-40.1	11	Lin et al. (2004)
8	Nipponbare/Kasalath//Nipponbare	BC_1F_9 to BC_1F_{12}	98	2.2-6.38	11293	16	Takehisa et al. (2004)
9	Nona Bokra/Koshihikari	BC_3F_2	192	11.74	40.1	1	Ren et al.(2005)
10	Jiucaiqing/IR36	F_2	250	2.06-2.73	6.7-19.3	7	Yao et al.(2005)
11	Milyang 23/Gihobyeo	RIL (F_{19})	164	3.42-11.58	9.16-27.76	2	Lee et al (2006)
12	Nipponbare/Kasalath//Nipponbare	BILs	98	3.5-31.7	30529	2	Takehisa et al. (2006)
13	CSR 27/MI48	F_2:F_3	200	2.24-2.8	5.13-14.38	6	Ammar et al. (2007)
14	Tarommahalli/Khazar	F_2:F_3	192	2.65-5.54	9.03-38.22	32	Sabouri and Sabouri (2008)
15	IR64/Binam	BC_2F_8 Ils	99	--	--	35	Zang et al. (2008)
16	TN1/CJ06	DH	120	--	4.15-14.85	14	Cheng et al.(2008)
17	Ilpumbyeo/Moroberekan	BC_3F_5-ILs	117	2.1-3.2	10.2-13.9	6	Kim et al. (2009)
18	CSR27/ MI48	F2&F3 families	200	2.17-10.08	11.13-55.72	25	Ammar et al. (2009)
19	Tarommahalli/Khazar	F_2:F_3	192	12.35-24.51	9.7-20.9	14	Sabouri et al. (2009)
20	R29/Pokkali	RILs	140	2.5-11.3	24624	27	Thomson et al. (2010)
21	CSR 27/MI48	F_7 RILs	216	2.94-4.43	5.06-14.05	9	Pandit et al. (2010)
22	IR 29/ Pokkali	BC_3F_4	181	3.1-12.47	4-18.42	13	Alam et al. (2011)
23	Tarome-Molaei*3/ Tiqing	BC_2F_5 BILs	62	3-7.8	11202	14	Ahmadi and Fotokian (2011)
24	Teqing/Oryza rufipogon Accession	ILs	87		46235	15	Tian et al. (2011)

25	Shaheen Basmati,/Pokkali	$F_{2:3}$	190		5.27-10.55	22	Javed et al. (2011)
26	BRRI dhan40/IR61920-3B-22-2-1	F_2	300	2.7-7	12.5-29	3	Islam et al. (2011)
27	IR26/Jiucaiqing	$F_{2:9}$ RILS	150	3.5-26.6	6.5-43.7	7	Wang et al. (2011)
28	IR 26/Jiucaiqing	$F_{2:9}$ RILS	150	2.9-6.9	7.8-23.9	9	Wang et al.(2012)
29	Gharib/Sepidroud	F_2:F_4	148	2.53-4.19	0.02-29.71	41	Ghomi et al. (2013)
30	Sadri/FL478	F_2	232	2.2-18	5.2-30	37	Mohammadi et al.(2013)
31	IR29/Hasawi	F_5 RILS	300	3.4-17.1	10.6-42.3	7	Bimpong et al. (2013)
32	Gharib/Sepidroud	F_2:F_4	148	2.7-4.48	10.03-21.9	17	Mardani et al. (2014)
33	Dongnong425/Changbai10	$BC_2F_{2:3}$	190	2.58-4.13	8.4-16.6	13	Zheng et al. (2015)
34	IR64/IR4630-22-2-5-1-3	F_2	201	2.65-15.31	0.22-76.02	40	Calapit-Palao et al. (2015)
35	Pusa Basmati 1/Cheriviruppu	F_2	218	3-22.3	3.8-48.7	24	Hossain et al. (2015)
36	Ce258/IR75862-206-2-8-3-B-B-B(Zhongguangxiang1/IR75862-206-2-8-3-B-B-B)	BC_1F_{10}	400	2.5-10.04	5.13-13.75	18	Qiu et al. (2015)
37	Bg352/At354	F_5 RILs	281	2.7-12.86	12.4-46.7	83	Gimhani et al. (2016)
38	Bengal/ Pokkali	F_6 RILs	187	2.01-36.91	2.05-51.64	100	De Leon et al.(2016)
39	TCCP266/ Sakha102	F_2	288	2.05-3.95	5.3-11.2	10	Fayed and Farid, (2017)
40	93-11/O. rufipogon accession	BC_3ILs	285	44257	44410	16	Wang et al.(2017)
41	Bengal/ Pokkali	AB-Ils:BC_4F_4	292	2.03-6.31	2.64-27.93	50	De Leon et al.(2017)
42	IR29/Hasawi	$F_{5:6}$ RILs	142	3.1-20.6	5.4-48.4	34	Rahman et al. (2017)
43	IR29/Hasawi	F_5 RILs	142	3.06-7.21	9.3-20.6	20	Bizimana et al.2017
44	Jupiter/Nona Bokra	BC_3F_4 ILs	138	2.01-13.66	0.7-12.5	33	Puram et al.(2017)
45	At354 / Bg352	F_5 RILs	100	2.1-4.5	8.-16	6	Dahanayaka et al. (2017)
46	Cheniere/ Nona Bokra	BC_3F_4 ILs	112	2-9.9	1.2-17.6	50	Puram et al.(2018)
**Wheat**							
1	Opata 85 / W7984	RILs	114	>3	6.91-31.6	36	De Leon et al. (2011)
2	Roshan / Sabalan	RILs	254	2.8-5.3	43160	31	Ghaedrahmat et al. (2014)
3	WTSD91/ WN-64	F_2	154	2.5-29.1	0.01-18.79	49	Hussain et al. (2017)
4	Excalibur / Kukri	DH	233	3.6-12.9	8.1-28.2	6	Asif et al. (2018)
5	Xiaoyan 54 / Jing 411	RILs	142	3.0-3.8	8.3-19	6	Ren et al. (2018)
6	Excalibur /Kukri	RILs	128	3.4-3.8	8.7-10.9	6	Asif et al. (2021)
**Barley**							

1	CM72 / Gairdner	DH	93	2.78-10.57	3.25 - 27.81	13	Xue et al. (2009)
2	TX9425 / Naso Nijo.	DH	188	24.37	45	1	Xu et al. (2012)
3	Yangsimai 1 (YSM1) / Gairdner	DH	175	3.35-7.78	9.1-18.4	8	Ma et al. (2015)
4	CM72/Gairdner	DH	103	3.01-7.67	7.60-29.0	13	Mwando et al. (2021)
**Chickpea**							
1	ICCV2 /JG11	RILs	184	2.0-9.6	6.0-20.3	20	Samineni et al. (2010)
2	JG 62/ ICCV 2	RILs	126	2.5-6.9	7.7-55.6	31	Vadez et al. (2012)
3	ICCV 2 / JG 1	RILs	188	2.52-43.2	6.13 - 67	46	Pushpavalli et al. (2015)
4	ICCV 10/DCP92-3	RILs	201	3.1-8.7	6.1-28.7	28	Soren et al. (2020)
**Soybean**							
1	S-100/ Tokyo	$F_{2:5}$	106	12.9	79	1	Lee et al. (2004)
2	Kefeng No. 1/ Nannong1138-2	RILs	184	2.8-5.0	7.1–19.7	8	Chen et al. (2008)
3	Jackson/JWS156-1	F_2	255	43.4	68.7	1	Hamweih and Xu (2008)
4	Jackson/ JWS156-1	F_2 &F_6	112,149	4.0-14.5	13.0% -50.2	1	Tuyen et al. (2010)
5	FT-Abyara/C01; Jin dou No. 6 / 0197	RILs	96 & 81	--	44-47.1	1	Hamweih et al. (2011)
6	PI483463 / Hutcheson	RILs	106	18.8	56.5	1	Ha et al. (2013)
7	Kefeng1 / Nannong1138-2	RILs	184	2.12-7.19	4.49-25.94	11	Kan et al. (2016)
8	Jidou 12/ Ji NF 58	RILs	156	20.1	44.7	1	Shi et al. (2018)

Table S2. Genes used for salt stress tolerance development in plants using genetic engineering/transgenic approach.

S.No.	Gene Name	Gene Source	Promoter	Recipient genotype	Phenotype	References
**Kinases**						
1	*OsMAPK5*	*Oryza sativa*	CaMV35S	*Nipponbare (japonica rice)*	Over-expression of the gene:Increased tolerance to drought, salt, and cold stresses	Xiong and Yang (2003)
					Suppression of the gene: Enhanced resistance to fungal *(Magnaporthe grisea)* and bacterial *(Burkholderia glumae)* pathogens (dsRNAi transgenic plants)	
2	*OsMAPK44*	*Oryza sativa*	-	*Dongjin (japonica rice)*	Less damage and greater ratio of potassium and sodium (salinity stress) in overexpression transgenic lines	Jeong et al. (2006)
					21% more chlorophyll content in leaves of RNAi transgenic lines	
3	*OsMAPK33*	*Oryza sativa*	CaMV35S	*Dongjin (japonica rice), Vandana and IR64 (indica rice)*	Over-expression of the gene: Greater reduction in biomass accumulation	Lee et al. (2011)
					Higher sodium uptake into cells, resulting in a lower K^+/Na^+ ratio inside the cell	
4	*OsMKK6*	*Oryza sativa*	CaMV35S	*Pusa Basmati-1 (indica rice)*	Over-expression of the gene increased root/shoot length and weight in200 mM NaCl solution	Kumar and Sinha (2013)
					Less chlorophyll bleaching and higher MAPK activity	
5	*OsMKK1*	*Oryza sativa*	CaMV35S	*Dongjin (japonica rice)*	OsMKK1-knockout (osmkk1) mutant was more sensitive to salt stress ; Over-expression of OsMKK1 increased OsMPK4 activity in protoplasts	Wang et al. (2014)
6	*SIT1*	*Oryza sativa*	Ubiquitin promoter (*Zea mays*)	*Nipponbare (japonica rice)*	SIT1 negatively regulates salt tolerance in Rice; RNAi silencing of the gene significantly enhanced survival rates in salt stress	Li et al. (2014)
7	*OsCPK12*	*Oryza sativa*	CaMV35S	*Nipponbare (japonica rice)*	Over-expression of the gene increased tolerance to salt stress	Asano et al. (2012)
					The accumulation of hydrogen peroxide (H_2O_2) in the leaves reduced in overexpression lines	

8	*AtCIPK16*	*Arabidopsis thaliana*	CaMV35S	*Arabidopsis thaliana*	Over-expression of the gene enhanced shoot Na^+ exclusion<br>Increased biomass<br>Over-expression of the gene improved plant salinity tolerance	Amarasinghe et al. (2020)
9	*STRK1*	*Oryza sativa (Kitaake)*	CaMV35S	*Kitaake (japonica rice)*	Over-expression of the gene gives higher catalase activity and lower accumulation of H_2O_2<br>Higher tolerance to salt and oxidative stress	Zhou et al. (2018)
10	*SAPK4*	*Festuca rubra ssp. litoralis (red fescue)*	CaMV35S	*IR29 (indica rice)*	Over-expression of the gene improved germination, growth, and development under salt stress both in seedlings and mature plants<br>Less accumulation of Na^+ and Cl^- and improved photosynthesis	Diédhiou et al. (2008)
11	*OSRK1*	*Oryza sativa*	CaMV35S	*Nagdong (japonica rice)*	Over-expression of the gene: Seedling growth of the transgenic rice was retarded; Root elongation of OSRK1 transgenic rice was more sensitive to NaCl stress	Nam et al. (2012)
12	*OsSIK1*	*Oryza sativa*	CaMV35S	*TP309 (japonica rice)*	Over-expression of the gene: Tolerance to salt and drought stresses<br>Enhanced activities of peroxidase, superoxide dismutase and catalase<br>Less accumulation of H_2O_2 in leaves<br>Increased stomatal density of the leaf surface (adaxial and abaxial epidermis)	Ouyang et al. (2010)
13	*OsSIK2*	*Oryza sativa*	CaMV35S	*TP309 and Nipponbare (japonica rice)*	Over-expression of the gene: Tolerance to salt and drought stress<br>Early leaf development and a delayed dark induced senescence phenotype	Chen et al. (2013)
**Transcription Factors**						
14	*SNAC1*	*Oryza sativa*	CaMV35S	*Nipponbare (japonica rice)*	Over-expression of the gene: significant improvement in drought resistance and salt tolerance at the vegetative stage<br>Significantly enhances drought resistance (22–34% higher)	Hu et al. (2006)

15	*Rab7*	*Oryza sativa*	Ubiquitin promoter *(Zea mays)*	*Zhonghua (japonica rice)*	Over-expression of the gene: Enhanced seedling growth and increased proline content under salt-treated conditions<br>Increased number of vesicles in the root tip	Peng et al. (2014)
16	*OsNAC6*	*Oryza sativa*	OsNAC6	*Nipponbare (japonica rice)*	Over-expression of the gene led to growth retardation and low reproductive yields<br>Improved tolerance to dehydration and high-salt stresses<br>Increased tolerance to blast disease	Nakashima et al. (2007)
17	*OsDREB1F*	*Oryza sativa cv. IAPAR9*	CaMV35S	*Nipponbare (japonica rice)*	Over-expression of the gene: enhanced tolerance to salt, drought, and low temperature	Wang et al. (2008)
18	*OsDREB2A*	*Oryza sativa (Nipponbare)*	4ABRC (stress-inducible promoter)	*TNG67 (japonica rice)*	Over-expression of the gene: improved survival rates under severe drought and salt stress conditions<br>Increased number of developed longer roots	Cui et al. (2011)
19	*OsDREB2A*	*Oryza sativa (Pokkali)*	rd29A (stress inducible promoter)	*MTU 7029 (indica rice)*	Over-expression of the gene: tolerance to osmotic, salt and dehydration stresses	Mallikarjuna et al. (2011)
20	*GmDREB6*	*Glycine max*	CaMV35S	*Glycine max*	Over-expression of the gene: enhances proline accumulation and salt tolerance	Nguyen et al. (2019b)
21	*GmDREB1*	*Glycine max*	Ubiquitin promoter *(Zea mays)*	*Triticum aestivum L.*	Overexpression of the gene improves salt tolerance in transgenic wheat; Longer coleoptiles and radicles and a greater radical number at the germination stage<br>Higher root length, fresh weight, and tiller number per plant at the seedling stage<br>Up-regulation of osmotic and oxidative-stress related proteins<br>Higher levels of proline and betaine<br>Lower levels of malondialdehyde and relative electrolyte leakage	Jiang et al. (2014)
22	*OsbZIP23*	*Oryza sativa cv. IAPAR9*	Ubiquitin promoter *(Zea mays)*	*IRAT109 (japonica rice)*	Over-expression of the gene improved tolerance to drought and high-salinity stresses and sensitivity to ABA	Xiang et al. (2008)

23	*Scdr1*	*Saccharum officinarum*	CaMV35S	*Nicotiana tabacum*	Over-expression of the gene increased tolerance to drought, salinity and oxidative stress<br>Increased photosynthesis, water content, biomass, germination rate and chlorophyll content. Reduced accumulation of ROS	Begcy et al. (2012)
24	*OsABI5*	*Oryza sativa*	CaMV35S	*Nongken 58 (japonica rice)*	Suppression of the gene promoted stress resistance to NaCl and PEG	Zou et al. (2008)
25	*DST*	*Oryza sativa*	CaMV35S	*Zhonghua 11 (japonica rice)*	Knockout of the gene using RNAi or mutagenesis increased stomatal closure and reduces stomatal density, consequently resulting in enhanced drought and salt tolerance in rice	Huang et al. (2009)
26	*ONAC045*	*Oryza sativa (Guangluai 4)*		*Nipponbare (japonica rice)*	Over-expression of the gene enhanced tolerance to drought and salt treatments	Zheng et al. (2009)
27	*OsMYB2*	*Oryza sativa*	CaMV35S	*Zhonghua 10 (japonica rice)*	Over-expression of the gene led to more tolerant to salt, cold, and dehydration stresses and more sensitive to abscisic acid<br>Accumulated greater amounts of soluble sugars and proline<br>Enhanced up-regulation of the genes encoding proline synthase and transporters<br>Accumulated less amounts of H_2O_2 and malondialdehyde	Yang et al. (2012)
28	*OsTZF1*	*Oryza sativa*	Ubiquitin promoter *(Zea mays)*	*Nipponbare (japonica rice)*	Over-expression of the gene delayed seed germination, growth retardation at the seedling stage, and delayed leaf senescence<br>Improved tolerance to high-salt and drought stresses	Jan et al. (2013)
29	*SERF1*	*Oryza sativa*	Ubiquitin promoter *(Zea mays)*	*Nipponbare (japonica rice)*	Constituttive expression of the gene improves salinity tolerance	Schmidt et al. (2013)

30	*OsNAC022*	*Oryza sativa*	Ubiquitin promoter *(Zea mays)*	*Yuanfengzao (indica rice)*	Over-expression of the gene increased drought tolerance, leading to higher survival ratios and better growth	Hong et al. (2016)
					Enhanced salt tolerance and accumulated less Na^+ in roots and shoots	
					Decreased rates of water loss and transpiration, reduced percentage of open stomata and increased contents of proline and soluble sugars.	
					Increased sensitivity to exogenous ABA at seed germination and seedling growth stages but contained higher level of endogenous ABA	
31	*OsSPL10*	*Oryza sativa*	U6 promoter *(Oryza sativa)*	*R401 (indica rice)*	Knockout of the genes using genome editing enhanced salt tolerance and glabrous leaves and glumes	Lan et al. (2019)
32	*OsHsfA7*	*Oryza sativa*	CaMV35S	*Nipponbare (japonica rice)*	Over-expression of the gene increased tolerance to salt and drought stresses	Liu et al. (2013)
33	*ZFP179*	*Oryza sativa*	CaMV35S	*Jiucaiqing (japonica rice)*	Over-expression of the gene increased salt tolerance and hypersensitivity to exogenous ABA	Sun et al. (2010)
					Increased levels of free proline and soluble sugars	
					Significantly increased tolerance to oxidative stress, the reactive oxygen species (ROS)-scavenging ability	
					Significantly increased expression levels of stress-related genes (OsDREB2A, OsP5CS OsProT, and OsLea3) under salt stress	
34	*OsGATA8*	*Oryza sativa*	CaMV35S	*IR64 and Pokkali (indica rice)*	Over-expression of the gene enhanced biomass accumulation and higher photosynthetic efficiency under both normal and salinity-stress conditions; ~46% higher yield under stress	Nutan et al. (2019)
35	*OsMYB6*	*Oryza sativa*	CaMV35S	*Zhonghua 11 (japonica rice)*	Over-expression of the gene increased tolerance to drought and salt stress	Tang et al. (2019)
					Higher proline content	
					Higher CAT and SOD activities	
					Lower REL and MDA content	

36	*OsNAC45*	*Oryza sativa*	Ubiquitin promoter *(Zea mays)*	*Nipponbare (japonica rice)*	Knockout of the gene by genome editing led to more ROS accumulation in roots and increased sensitivity of rice to salt stress	Zhang et al. (2020)
37	*GhWRKY5*	*Gossypium aridum L.*		*Arabidopsis thaliana*	Over-expression of the gene enhanced salt tolerance at the stages of seed germination and vegetative growth.	Guo et al. (2019)
					Higher activities of superoxide dismutase (SOD) and peroxidase (POD)	
**MicroRNAs**						
38	*osa-MIR393*	*Oryza sativa*	CaMV35S	*Kongyu 131 (japonica rice)*	Over-expression of miRNA made the plants more sensitive to salt and alkali treatment	Gao et al. (2011)
39	*OsmiR393*	*Oryza sativa*	CaMV35S	*Zhonghua 11 (japonica rice)*	Over-expression if miRNA led to increased tillers and early flowering	Xia et al. (2012)
					Reduced tolerance to salt and drought and hyposensitivity to auxin	
					The expression of an auxin transporter (OsAUX1) and a tillering inhibitor (OsTB1) were down regulated	
**Helicases**						
40	*SUV3*	*Oryza sativa*	CaMV35S	*IR64 (indica rice)*	Over-expression of the gene led to significantly higher endogenous content of plant hormones viz., gibberellic acid (GA3), zeatin (Z) and indole-3-acetic acid (IAA) in leaf, stem and root under salt stress	Sahoo et al. (2014)
41	*OsRuvB*	*Oryza sativa*	CaMV35S	*Cajanus cajan*	In planta expression of the gene led to increase in chlorophyll content, relative water content, peroxidase and catalase activity under salinity stress	Singh et al. (2020)
					Significant reduction in membrane injury index and lipid peroxidation	
**Na^+/H^+ antiporter/exchanger and Na^+/K^+ symporter**						
42	*OsNHX1*	*Oryza sativa (Pokkali)*	CaMV35S	*Pokkali (indica rice)*	Over-expression of the gene improves salt stress tolerant	Amin et al. (2016)

43	*SOS1 and AHA*	*Sesuvium portulacastrum*	CaMV35S	*Arabidopsis thaliana*	Plants expressing both SpSOS1 and SpAHA1 grew better under salt stress; higher Na^+ and H^+ efflux rates<br>Lower malondialdehyde (MDA)<br>Highest K^+ level	Fan et al. (2019)
44	*OsHKT1;4*	*Oryza sativa*	Ubiquitin promoter (*Zea mays*)	*Nipponbare (japonica rice)*	RNAi suppression of the gene: Significant Na+ over accumulation in aerial organs, in particular, leaf blades and sheaths in RNAi plants in the reproductive growth stage in salinity stress	Suzuki et al. (2016)
45	*AtHKT1*	*Arabidopsis thaliana*	CaMV35S	*Nicotiana tabacum L.*	Overexpression of the gene peivdes salinity tolerance, plants maintain healthy K+ status<br>Enhanced SOD, CAT and POD activities<br>Raised chlorophyll and soluble sugar contents and root activity<br>Decreased MDA and proline and electrolyte leakage destruction.	Wang et al. (2018)
46	*SOD2*	*Schizosaccharomyces pombe*	CaMV35S	*Arabidopsis thaliana*	Over-expression of the gene improved seed germination and seedling salt tolerance<br>Less accumulation of Na^+ and more K^+ in the symplast<br>Higher photosynthetic rate and the fresh weight after NaCl treatment.	Gao et al. (2003)
**Proline and glycine Metabolism**						
47	*P5CS*	*Vigna aconitifolia*	CaMV35S	*ADT 43 (indica rice)*	Over-expression of the gene improves salt stress tolerant	Karthikeyan et al. (2011)
48	*P5CS*	*Phaseolus vulgaris*	CaMV35S	*Arabidopsis thaliana*	Over-expression of the gene: Higher levels of PvP5CS1 and PvP5CS2 transcripts<br>Significantly higher ($P < 0.01$) proline content<br>Significantly lower ($P < 0.01$) relative electrical conductivity (REC)<br>Biomass production of transgenic lines was significantly higher ($P < 0.05$) in salt stress	Chen et al. (2013)
49	*SIDP361*	*Oryza sativa*	CaMV35S	*Taipei309 (japonica rice)*	Over-expression of the gene improved tolerance to salt stress at both the seedling and heading stages<br>Elevated amounts of free proline	Li et al. (2016)

50	*codA*	*Arthrobacter globiformis*		*Pusa Basmati 1 (indica rice)*	Over-expression of the gene: approximately 50% of the transgenic plants survive in salt stress and set seed whereas wild-type plants failed	Mohanty et al. (2002)
**Vacuolar Proteins**						
51	*AVP1*	*Arabidopsis thaliana*	Ubiquitin promoter *(Zea mays)*	*Dongjin (japonica rice)*	Over-expression of the gene significantly enhanced tolerance to salinity<br>Improved ion homoeostasis through increased accumulation of Na^+ ions in whole tissues, ~15% higher culm and root weights	Kim et al. (2014)
52	*SaVHAc1*	*Spartina alterniflora*	CaMV35S	*Cocodrie (japonica rice)*	Over-expression of the gene enhanced tolerance to salt stress<br>Increased accumulation cation transport and ABA signaling genes<br>Maintained higher relative water content under salt stress through early stage closure of the leaf stoma and reduced stomata density<br>Higher root and leaf growth and yield under salt stress	Baisakh et al. (2012)
53	*AtAVP1* and *AtNHX1*	*Arabidopsis thaliana*	CaMV35S	*Glycine max*	Co-expressing AtAVP1 and AtNHX1 enhanced salt tolerance<br>Higher chlorophyll content, cell membrane stability, and photosynthetic rate under salt stress.	Nguyen et al. (2019a)
**Gibberellin Metabolism**						
54	*GA2ox*	*Oryza sativa*	Ubiquitin promoter *(Zea mays)*	*Tainung 67 (japonica rice)*	Ectopic expression of the gene mutants: Moderately lowered GA levels<br>Reduced plant height<br>More productive tillers, expanded root system, higher WUE and photosynthesis rate<br>Elevated abiotic and biotic stress<br>Increased grain yield by 10–30% in field trials	Lo et al. (2017)

**Phospholipids Metabolism**						
55	*MIPS*	*Ipomoea batatas*	CaMV35S	*Ipomoea batatas*	Over-expression of the gene led to up-regulation of salt stress responsive genes (myo-inositol monophosphatase), pyrroline-5-carboxylate synthase, pyrroline-5-carboxylate reductase, psbA, phosphoribulokinase and superoxide dismutase) under salt stress	Wang et al. (2016)
**Hormone-like peptides**						
56	*AtPROPEP3*	*Arabidopsis thaliana*	CaMV35S	*Arabidopsis thaliana*	Over-expression of the gene conferred increased salinity stress; Inhibition of the salt induced bleaching of chlorophyll in seedlings	Nakaminami et al. (2018)
					Significant root length increment	
**Oxidases**						
57	*Bna.AOX1b*	*Brassica napus*	CaMV35S	*Brassica napus*	Over-expression of the gene improved seed germination under osmotic and salt stress	Yang et al. (2019)
**ROS-producing/scavenging enzyme**						
58	*PDH45*		CaMV35S	*IR64 (indica rice)*	Over-expressing transgenic plants accumulate lower levels of Na^+, ROS and maintain $[Ca^{2+}]$ cyt and exhibit higher cell viability	Nath et al. (2016)
**Calcium signaling**						
59	*OsCam1-1*	*Oryza sativa (FL530-IL)*	CaMV35S	*Khoa Dawk Mali 105 (indica rice)*	Over-expression of the gene led to more tolerance to salt stress and maintain their shoot and root biomass (as dry weight)	Saeng-ngam et al. (2011)
60	*OsCPK4*	*Oryza sativa*	Ubiquitin promoter *(Zea mays)*	*Nipponbare (japonica rice)*	Over-expression of the gene enhances tolerance to salt and drought stress	Campo et al. (2014)
					Stronger water-holding capability and reduced levels of membrane lipid peroxidation and electrolyte leakage under drought or salt stress conditions	
					Accumulate less Na^+ in their roots	

61	Calcineurin	Catalytic subunit of mouse calcineurin	CaMV35S	*Xiushui (japonica rice)*	over-expression of the gene imparts higher salt stress tolerance<br>Root growth was less inhibited than the shoot growth, and that less Na^+ was accumulated in the roots<br>Increased expression of the Rab16A gene that encodes a group 2-type late-embryogenesis abundant (LEA) protein	Ma et al. (2005)
**Trehalose biosynthesis**						
62	*OsTPS1*	*Oryza sativa ssp. indica (Guangluai 4)*	Actin1 promoter	*ZH 11 and Nipponbare (japonica rice)*	Over-expression of the gene improved tolerance to cold, high salinity and drought treatments<br>Higher concentrations of trehalose and proline and some stress-related genes were up-regulated, including WSI18, RAB16C, HSP70, and ELIP	Li et al. (2011)
**Aquaporin**						
63	*HvPIP2;1*	*Hordeum vulgare*	CaMV35S	*Kinuhikari (japonica rice)*	Over-expression of the gene induced better growth reduction under salt stress<br>Decrease in shoot water content (from 79% to 61%)<br>Reduction of root mass or shoot mass (both less than 40%)	Katsuhara et al. (2003)
**Brassinosteroid**						
64	*OsGSK1(BIN2)*	*Oryza sativa*	Promoter less reporter gene b-glucuronidase (GUS)	*Dongjin (japonica rice)*	T-DNA mutation of the gene led to enhanced tolerance to cold, heat, salt, and drought stresses (knockout plants)	Koh et al. (2007)
**Histone-gene binding protein**						
65	*OsHBP1b*	*Oryza sativa*	CaMV35S	*IR64 (indica rice)*	Over-expression of the gene provides better survival and favourable osmotic parameters under salinity stress<br>Improved tolerance in drought and high temperature with better root and shoot growth, photosynthetic parameters, and enhanced antioxidant enzyme activity	Das et al. (2019)

**Apoptosis**						
66	*SfIAP*	*Spodoptera frugiperda*	Ubiquitin promoter *(Zea mays)*	*Nipponbare (japonica rice)*	Over-expression of the gene improved salinity tolerance, retain plant water status, ion homeostasis, photosynthetic efficiency and growth to successfully combat salinity	Hoang et al. (2014)
**Calvin cycle of Phostosyntheis**						
67	*SBPase*	*Oryza sativa ssp. indica (cultivar 9311)*	Ubiquitin promoter *(Zea mays)*	*Zhonghua 11 (japonica rice)*	Over-expression of the gene enhanced tolerance to salt stress at the young seedlings stage<br>CO_2 assimilation, significantly more tolerant to salt stress	Feng et al. (2007)

Supplemental References:

Ahmadi J, Fotokian M-H (2011) Identification and mapping of quantitative trait loci associated with salinity tolerance in rice (Oryza sativa) using SSR markers. Iranian journal of biotechnology 9(1): 21-30

Alam R, Sazzadur Rahman M, Seraj ZI, Thomson MJ, Ismail AM, Tumimbang-Raiz E, Gregorio GB (2011) Investigation of seedling-stage salinity tolerance QTLs using backcross lines derived from Oryza sativa L. Pokkali. Plant Breeding 130(4): 430-437

Amarasinghe SL, Huang W, Watson-Haigh NS, Gilliham M, Roy SJ, Baumann U (2020) AtCIPK16 Mediates Salt Stress Potentially Through Phytohormones and Transcription Factors. bioRxiv: 2020.2002.2017.953216

Amin US, Biswas S, Elias SM, Razzaque S, Haque T, Malo R, Seraj ZI (2016) Enhanced Salt Tolerance Conferred by the Complete 2.3 kb cDNA of the Rice Vacuolar Na(+)/H(+) Antiporter Gene Compared to 1.9 kb Coding Region with 5' UTR in Transgenic Lines of Rice. Front Plant Sci 7: 14

Ammar M, Pandit A, Singh R, Sameena S, Chauhan M, Singh A, Sharma P, Gaikwad K, Sharma T, Mohapatra T (2009) Mapping of QTLs controlling Na+, K+ and CI− ion concentrations in salt tolerant indica rice variety CSR27. Journal of Plant Biochemistry and Biotechnology 18(2): 139-150

Ammar M, Singh R, Singh A, Mohapatra T, Sharma T, Singh N Mapping QTLs for salinity tolerance at seedling stage in rice (Oryza sativa L.). Proceedings of the African crop science conference proceedings, 2007, pp 617-620

Asano T, Hayashi N, Kobayashi M, Aoki N, Miyao A, Mitsuhara I, Ichikawa H, Komatsu S, Hirochika H, Kikuchi S, Ohsugi R (2012) A rice calcium-dependent protein kinase OsCPK12 oppositely modulates salt-stress tolerance and blast disease resistance. Plant J 69(1): 26-36

Begcy K, Mariano ED, Gentile A, Lembke CG, Zingaretti SM, Souza GM, Menossi M (2012) A novel stress-induced sugarcane gene confers tolerance to drought, salt and oxidative stress in transgenic tobacco plants. PLoS One 7(9): e44697

Bizimana JB, Luzi-Kihupi A, R WM, Singh RK (2017) Identification of quantitative trait loci for salinity tolerance in rice (Oryza sativa L.) using IR29/Hasawi mapping population. J Genet 96(4): 571-582

Bonilla P, Dvorak J, Mackell D, Deal K, Gregorio G (2002) RFLP and SSLP mapping of salinity tolerance genes in chromosome 1 of rice (Oryza sativa L.) using recombinant inbred lines. Philippine Agricultural Scientist (Philippines)

Calapit-Palao C. D. VCB, Thomson M. J., and Singh R.K. QTL identification for reproductive-stage salinity tolerance in rice *(Oryza sativa L.)*. Proceedings of the Proceedings of SABRAO 13th Congress and International Conference, September 14–16 2015, Bogor, Indonesia

Campo S, Baldrich P, Messeguer J, Lalanne E, Coca M, San Segundo B (2014) Overexpression of a Calcium-Dependent Protein Kinase Confers Salt and Drought Tolerance in Rice by Preventing Membrane Lipid Peroxidation. Plant Physiol 165(2): 688-704

Chen H, Cui S, Fu S, Gai J, Yu D (2008) Identification of quantitative trait loci associated with salt tolerance during seedling growth in soybean (Glycine max L.). Australian Journal of Agricultural Research 59(12): 1086-1091

Chen JB, Yang JW, Zhang ZY, Feng XF, Wang SM (2013) Two P5CS genes from common bean exhibiting different tolerance to salt stress in transgenic Arabidopsis. Journal of genetics 92(3): 461-469

Chen LJ, Wuriyanghan H, Zhang YQ, Duan KX, Chen HW, Li QT, Lu X, He SJ, Ma B, Zhang WK, Lin Q, Chen SY, Zhang JS (2013) An S-domain receptor-like kinase, OsSIK2, confers abiotic stress tolerance and delays dark-induced leaf senescence in rice. Plant Physiol 163(4): 1752-1765

CHENG H-T, JIANG H, Da-Wei X, Long-Biao G, Da-Li Z, ZHANG G-H, Qian Q (2008) Mapping of QTL underlying tolerance to alkali at germination and early seedling stages in rice. Acta Agronomica Sinica 34(10): 1719-1727

Claes B, Dekeyser R, Villarroel R, Van den Bulcke M, Bauw G, Van Montagu M, Caplan A (1990) Characterization of a rice gene showing organ-specific expression in response to salt stress and drought. The Plant Cell 2(1): 19-27

Cui M, Zhang W, Zhang Q, Xu Z, Zhu Z, Duan F, Wu R (2011) Induced over-expression of the transcription factor OsDREB2A improves drought tolerance in rice. Plant Physiol Biochem 49(12): 1384-1391

Das P, Lakra N, Nutan KK, Singla-Pareek SL, Pareek A (2019) A unique bZIP transcription factor imparting multiple stress tolerance in Rice. Rice (N Y) 12(1): 58

De Leon TB, Linscombe S, Subudhi PK (2016) Molecular dissection of seedling salinity tolerance in rice (Oryza sativa L.) using a high-density GBS-based SNP linkage map. Rice 9(1): 1-22

De Leon TB, Linscombe S, Subudhi PK (2017) Identification and validation of QTLs for seedling salinity tolerance in introgression lines of a salt tolerant rice landrace 'Pokkali'. PLoS One 12(4): e0175361

Diédhiou CJ, Popova OV, Dietz KJ, Golldack D (2008) The SNF1-type serine-threonine protein kinase SAPK4 regulates stress-responsive gene expression in rice. BMC Plant Biol 8: 49

Fayed AM, Farid MA (2017) Mapping of Quantitative Trait Loci (QTL) for Na+ and K+ Uptake Controlling Rice Salt Tolerance (Oryza sativa L.). Int J Curr Microbiol App Sci 6(1): 462-471

Feng L, Han Y, Liu G, An B, Yang J, Yang G, Li Y, Zhu Y (2007) Overexpression of sedoheptulose-1,7-bisphosphatase enhances photosynthesis and growth under salt stress in transgenic rice plants. Funct Plant Biol 34(9): 822-834

Gao P, Bai X, Yang L, Lv D, Pan X, Li Y, Cai H, Ji W, Chen Q, Zhu Y (2011) osa-MIR393: a salinity- and alkaline stress-related microRNA gene. Mol Biol Rep 38(1): 237-242

Gao X, Ren Z, Zhao Y, Zhang H (2003) Overexpression of SOD2 increases salt tolerance of Arabidopsis. Plant physiology 133(4): 1873-1881

Ghaedrahmati M, Mardi M, Naghavi M, Majidi Haravan E, Nakhoda B, Azadi A, Kazemi M (2018) Mapping QTLs associated with salt tolerance related traits in seedling stage of wheat (Triticum aestivum L.).

Ghomi K, Rabiei B, Sabouri H, Sabouri A (2013) Mapping QTLs for traits related to salinity tolerance at seedling stage of rice (Oryza sativa L.): an agrigenomics study of an Iranian rice population. Omics: a journal of integrative biology 17(5): 242-251

Gimhani DR, Gregorio GB, Kottearachchi NS, Samarasinghe WL (2016) SNP-based discovery of salinity-tolerant QTLs in a bi-parental population of rice (Oryza sativa). Mol Genet Genomics 291(6): 2081-2099

Guo Y, Qiu Q-S, Quintero FJ, Pardo JM, Ohta M, Zhang C, Schumaker KS, Zhu J-K (2004) Transgenic evaluation of activated mutant alleles of SOS2 reveals a critical requirement for its kinase activity and C-terminal regulatory domain for salt tolerance in Arabidopsis thaliana. The Plant Cell 16(2): 435-449

Ha B-K, Vuong TD, Velusamy V, Nguyen HT, Shannon JG, Lee J-D (2013) Genetic mapping of quantitative trait loci conditioning salt tolerance in wild soybean (Glycine soja) PI 483463. Euphytica 193(1): 79-88

Hamwieh A, Tuyen D, Cong H, Benitez E, Takahashi R, Xu D (2011) Identification and validation of a major QTL for salt tolerance in soybean. Euphytica 179(3): 451-459

Hamwieh A, Xu D (2008) Conserved salt tolerance quantitative trait locus (QTL) in wild and cultivated soybeans. Breeding Science 58(4): 355-359

Hoang TML, Williams B, Khanna H, Dale J, Mundree SG (2014) Physiological basis of salt stress tolerance in rice expressing the antiapoptotic gene SfIAP. Funct Plant Biol 41(11): 1168-1177

Hong Y, Zhang H, Huang L, Li D, Song F (2016) Overexpression of a Stress-Responsive NAC Transcription Factor Gene ONAC022 Improves Drought and Salt Tolerance in Rice. Front Plant Sci 7: 4

Hu H, Dai M, Yao J, Xiao B, Li X, Zhang Q, Xiong L (2006) Overexpressing a NAM, ATAF, and CUC (NAC) transcription factor enhances drought resistance and salt tolerance in rice. Proc Natl Acad Sci U S A 103(35): 12987-12992

Huang XY, Chao DY, Gao JP, Zhu MZ, Shi M, Lin HX (2009) A previously unknown zinc finger protein, DST, regulates drought and salt tolerance in rice via stomatal aperture control. Genes Dev 23(15): 1805-1817

Islam M, Hassan L, Salam M, Collard B, Singh R, Gregorio G (2011) QTL mapping for salinity tolerance at seedling stage in rice. Emirates Journal of Food and Agriculture: 137-146

Jan A, Maruyama K, Todaka D, Kidokoro S, Abo M, Yoshimura E, Shinozaki K, Nakashima K, Yamaguchi-Shinozaki K (2013) OsTZF1, a CCCH-tandem zinc finger protein, confers delayed senescence and stress tolerance in rice by regulating stress-related genes. Plant Physiol 161(3): 1202-1216

Javed MA, Huyop FZ, Wagiran A, Salleh FM (2011) Identification of QTLs for morph-physiological traits related to salinity tolerance at seedling stage in indica rice. Procedia Environmental Sciences 8: 389-395

Jeong M-J LS-K, Kim B-G, Kwon T-R, Cho W-S, Park Y-T, Lee J-O, Kwon H-B, Byun M-O, Park S-C (2006) A rice (Oryza sativa L.) MAP kinase gene, OsMAPK44, is involved in response to abiotic stresses. Plant Cell, Tissue and Organ Culture 85 (2): 151-160

Kan G, Ning L, Li Y, Hu Z, Zhang W, He X, Yu D (2016) Identification of novel loci for salt stress at the seed germination stage in soybean. Breeding science: 15147

Karthikeyan A PS, Ramesh M (2011) Transgenic indica rice cv. ADT 43 expressing a Δ1-pyrroline-5-carboxylate synthetase (P5CS) gene from Vigna aconitifolia demonstrates salt tolerance. . Plant Cell, Tissue and Organ Culture (PCTOC) 107(3): 383-395

Katsuhara M, Koshio K, Shibasaka M, Hayashi Y, Hayakawa T, Kasamo K (2003) Over-expression of a barley aquaporin increased the shoot/root ratio and raised salt sensitivity in transgenic rice plants. Plant Cell Physiol 44(12): 1378-1383

Kim D-M, Ju H-G, Kwon T-R, Oh C-S, Ahn S-N (2009) Mapping QTLs for salt tolerance in an introgression line population between japonica cultivars in rice. Journal of Crop Science and Biotechnology 12(3): 121

Kim Y KI-S, Choe Y, Bae M-J, Shin S-Y, Park S, Kang H-G, Kim YH, Yoon H (2014) Overexpression of the Arabidopsis vacuolar H + -pyrophosphatase AVP1 gene in rice plants improves grain yield under paddy field conditions. . The Journal of Agricultural Science 152: 941-953

Koh S, Lee SC, Kim MK, Koh JH, Lee S, An G, Choe S, Kim SR (2007) T-DNA tagged knockout mutation of rice OsGSK1, an orthologue of Arabidopsis BIN2, with enhanced tolerance to various abiotic stresses. Plant Mol Biol 65(4): 453-466

Koyama ML, Levesley A, Koebner RM, Flowers TJ, Yeo AR (2001) Quantitative trait loci for component physiological traits determining salt tolerance in rice. Plant physiology 125(1): 406-422

Kumar K, Sinha AK (2013) Overexpression of constitutively active mitogen activated protein kinase kinase 6 enhances tolerance to salt stress in rice. Rice (N Y) 6(1): 25

Lee SK, Kim BG, Kwon TR, Jeong MJ, Park SR, Lee JW, Byun MO, Kwon HB, Matthews BF, Hong CB, Park SC (2011) Overexpression of the mitogen-activated protein kinase gene OsMAPK33 enhances sensitivity to salt stress in rice (Oryza sativa L.). J Biosci 36(1): 139-151

Lee SY, Ahn JH, Cha YS, Yun DW, Lee MC, Ko JC, Lee KS, Eun MY (2006) Mapping of quantitative trait loci for salt tolerance at the seedling stage in rice. Molecules & Cells (Springer Science & Business Media BV) 21(2)

Li CH, Wang G, Zhao JL, Zhang LQ, Ai LF, Han YF, Sun DY, Zhang SW, Sun Y (2014) The Receptor-Like Kinase SIT1 Mediates Salt Sensitivity by Activating MAPK3/6 and Regulating Ethylene Homeostasis in Rice. Plant Cell 26(6): 2538-2553

Li HW, Zang BS, Deng XW, Wang XP (2011) Overexpression of the trehalose-6-phosphate synthase gene OsTPS1 enhances abiotic stress tolerance in rice. Planta 234(5): 1007-1018

Li M GL, Guo C, Wang L, Chen L (2016) Over-expression of a DUF1644 protein gene, SIDP361, enhances tolerance to salt stress in transgenic rice. Journal of Plant Biology 59(1): 62-73

Lin H, Zhu M, Yano M, Gao J, Liang Z, Su W, Hu X, Ren Z, Chao D (2004) QTLs for Na+ and K+ uptake of the shoots and roots controlling rice salt tolerance. Theoretical and Applied Genetics 108(2): 253-260

Liu AL, Zou J, Liu CF, Zhou XY, Zhang XW, Luo GY, Chen XB (2013) Over-expression of OsHsfA7 enhanced salt and drought tolerance in transgenic rice. BMB Rep 46(1): 31-36

Lo SF, Ho TD, Liu YL, Jiang MJ, Hsieh KT, Chen KT, Yu LC, Lee MH, Chen CY, Huang TP, Kojima M, Sakakibara H, Chen LJ, Yu SM (2017) Ectopic expression of specific GA2 oxidase mutants promotes yield and stress tolerance in rice. Plant Biotechnol J 15(7): 850-864

Ma X, Qian Q, Zhu D (2005) Expression of a calcineurin gene improves salt stress tolerance in transgenic rice. Plant Mol Biol 58(4): 483-495

Mallikarjuna G, Mallikarjuna K, Reddy MK, Kaul T (2011) Expression of OsDREB2A transcription factor confers enhanced dehydration and salt stress tolerance in rice (Oryza sativa L.). Biotechnol Lett 33(8): 1689-1697

Mardani Z, Rabiei B, Sabouri H, Sabouri A (2014) Identification of molecular markers linked to salt-tolerant genes at germination stage of rice. Plant Breeding 133(2): 196-202

Mohammadi R, Mendioro MS, Diaz GQ, Gregorio GB, Singh RK (2013) Mapping quantitative trait loci associated with yield and yield components under reproductive stage salinity stress in rice (Oryza sativa L.). Journal of genetics 92(3): 433-443

Mohanty A, Kathuria H, Ferjani A, Sakamoto A, Mohanty P, Murata N, Tyagi AK (2002) Transgenics of an elite indica rice variety Pusa Basmati 1 harbouring the codA gene are highly tolerant to salt stress. Theor Appl Genet 106(1): 51-57

Nakaminami K, Okamoto M, Higuchi-Takeuchi M, Yoshizumi T, Yamaguchi Y, Fukao Y, Shimizu M, Ohashi C, Tanaka M, Matsui M, Shinozaki K, Seki M, Hanada K (2018) AtPep3 is a hormone-like peptide that plays a role in the salinity stress tolerance of plants. Proceedings of the National Academy of Sciences 115(22): 5810-5815

Nakashima K, Tran LS, Van Nguyen D, Fujita M, Maruyama K, Todaka D, Ito Y, Hayashi N, Shinozaki K, Yamaguchi-Shinozaki K (2007) Functional analysis of a NAC-type transcription factor OsNAC6 involved in abiotic and biotic stress-responsive gene expression in rice. Plant J 51(4): 617-630

Nam MH, Huh SM, Kim KM, Park WJ, Seo JB, Cho K, Kim DY, Kim BG, Yoon IS (2012) Comparative proteomic analysis of early salt stress-responsive proteins in roots of SnRK2 transgenic rice. Proteome Sci 10: 25

Nath M, Yadav S, Kumar Sahoo R, Passricha N, Tuteja R, Tuteja N (2016) PDH45 transgenic rice maintain cell viability through lower accumulation of Na(+), ROS and calcium homeostasis in roots under salinity stress. J Plant Physiol 191: 1-11

Nguyen NT, Vu HT, Nguyen TT, Nguyen L-AT, Nguyen M-CD, Hoang KL, Nguyen KT, Quach TN (2019) Co-expression of Arabidopsis AtAVP1 and AtNHX1 to Improve Salt Tolerance in Soybean. Crop Science 59(3): 1133-1143

Niones JM (2004) Five mapping of the salinity tolerance gene on chromosome 1 of rice (Oryza sativa L.) using near-isogenic lines.

Nutan KK, Singla-Pareek SL, Pareek A (2020) The Saltol QTL-localized transcription factor OsGATA8 plays an important role in stress tolerance and seed development in Arabidopsis and rice. J Exp Bot 71(2): 684-698

Ouyang SQ, Liu YF, Liu P, Lei G, He SJ, Ma B, Zhang WK, Zhang JS, Chen SY (2010) Receptor-like kinase OsSIK1 improves drought and salt stress tolerance in rice (Oryza sativa) plants. Plant J 62(2): 316-329

Pandit A, Rai V, Bal S, Sinha S, Kumar V, Chauhan M, Gautam RK, Singh R, Sharma PC, Singh AK (2010) Combining QTL mapping and transcriptome profiling of bulked RILs for identification of functional polymorphism for salt tolerance genes in rice (Oryza sativa L.). Molecular Genetics and Genomics 284(2): 121-136

Peng X, Ding X, Chang T, Wang Z, Liu R, Zeng X, Cai Y, Zhu Y (2014) Overexpression of a Vesicle Trafficking Gene, OsRab7, enhances salt tolerance in rice. ScientificWorldJournal 2014: 483526

Puram VRR, Ontoy J, Linscombe S, Subudhi PK (2017) Genetic dissection of seedling stage salinity tolerance in rice using introgression lines of a salt tolerant landrace Nona Bokra. Journal of Heredity 108(6): 658-670

Puram VRR, Ontoy J, Subudhi PK (2018) Identification of QTLs for salt tolerance traits and prebreeding lines with enhanced salt tolerance in an introgression line population of rice. Plant Molecular Biology Reporter 36(5): 695-709

Qiu X, Yuan Z, Liu H, Xiang X, Yang L, He W, Du B, Ye G, Xu J, Xing D (2015) Identification of salt tolerance-improving quantitative trait loci alleles from a salt-susceptible rice breeding line by introgression breeding. Plant Breeding 134(6): 653-660

Rahman MA, Bimpong IK, Bizimana J, Pascual ED, Arceta M, Swamy BM, Diaw F, Rahman MS, Singh R (2017) Mapping QTLs using a novel source of salinity tolerance from Hasawi and their interaction with environments in rice. Rice 10(1): 1-17

Sabouri H, Rezai A, Moumeni A, Kavousi A, Katouzi M, Sabouri A (2009) QTLs mapping of physiological traits related to salt tolerance in young rice seedlings. Biologia Plantarum 53(4): 657-662

Sabouri H, Sabouri A (2008) New evidence of QTLs attributed to salinity tolerance in rice. African Journal of Biotechnology 7(24)

Saeng-ngam S TW, Buaboocha T, Chadchawan S (2011) The role of the OsCam1-1 salt stress sensor in ABA accumulation and salt tolerance in rice. . Journal of Plant Biology 55: 198-208

Sahoo RK, Ansari MW, Tuteja R, Tuteja N (2014) OsSUV3 transgenic rice maintains higher endogenous levels of plant hormones that mitigates adverse effects of salinity and sustains crop productivity. Rice (N Y) 7(1): 17

Samineni S (2010) Physiology, genetics and QTL mapping of salt tolerance in chickpea (Cicer arietinum L.). University of Western Australia,

Shi X, Yan L, Yang C, Yan W, Moseley DO, Wang T, Liu B, Di R, Chen P, Zhang M (2018) Identification of a major quantitative trait locus underlying salt tolerance in 'Jidou 12'soybean cultivar. BMC research notes 11(1): 1-6

Singh R SS, Kharb P, Saifi S and Tuteja N (2020) OsRuvB transgene induces salt tolerance in pigeon pea. Journal of Plant Interactions 15(1): 17-26

Sun SJ, Guo SQ, Yang X, Bao YM, Tang HJ, Sun H, Huang J, Zhang HS (2010) Functional analysis of a novel Cys2/His2-type zinc finger protein involved in salt tolerance in rice. J Exp Bot 61(10): 2807-2818

Suzuki K, Yamaji N, Costa A, Okuma E, Kobayashi NI, Kashiwagi T, Katsuhara M, Wang C, Tanoi K, Murata Y, Schroeder JI, Ma JF, Horie T (2016) OsHKT1;4-mediated Na(+) transport in stems contributes to Na(+) exclusion from leaf blades of rice at the reproductive growth stage upon salt stress. BMC Plant Biol 16: 22

Takehisa H, Shimodate T, Fukuta Y, Ueda T, Yano M, Yamaya T, Kameya T, Sato T (2004) Identification of quantitative trait loci for plant growth of rice in paddy field flooded with salt water. Field Crops Research 89(1): 85-95

Takehisa H, Ueda T, Fukuta Y, Obara M, Abe T, Yano M, Yamaya T, Kameya T, Higashitani A, Sato T (2006) Epistatic interaction of QTLs controlling leaf bronzing in rice (Oryza sativa L.) grown in a saline paddy field. Breeding Science 56(3): 287-293

Tian L, Tan L, Liu F, Cai H, Sun C (2011) Identification of quantitative trait loci associated with salt tolerance at seedling stage from Oryza rufipogon. Journal of Genetics and Genomics 38(12): 593-601

Tuyen DD, Lal S, Xu D (2010) Identification of a major QTL allele from wild soybean (Glycine soja Sieb. & Zucc.) for increasing alkaline salt tolerance in soybean. Theoretical and Applied Genetics 121(2): 229-236

Vadez V, Krishnamurthy L, Thudi M, Anuradha C, Colmer TD, Turner NC, Siddique KH, Gaur PM, Varshney RK (2012) Assessment of ICCV 2× JG 62 chickpea progenies shows sensitivity of reproduction to salt stress and reveals QTL for seed yield and yield components. Molecular Breeding 30(1): 9-21

Wang F-b, Zhai H, An Y-y, Si Z-z, He S-z, Liu Q-c (2016) Overexpression of IbMIPS1 gene enhances salt tolerance in transgenic sweetpotato. Journal of Integrative Agriculture 15(2): 271-281

Wang Q, Guan Y, Wu Y, Chen H, Chen F, Chu C (2008) Overexpression of a rice OsDREB1F gene increases salt, drought, and low temperature tolerance in both Arabidopsis and rice. Plant Mol Biol 67(6): 589-602

Wang S, Cao M, Ma X, Chen W, Zhao J, Sun C, Tan L, Liu F (2017) Integrated RNA sequencing and QTL mapping to identify candidate genes from Oryza rufipogon associated with salt tolerance at the seedling stage. Frontiers in plant science 8: 1427

Wang Z, Wang J, Bao Y, Wu Y, Zhang H (2011) Quantitative trait loci controlling rice seed germination under salt stress. Euphytica 178(3): 297-307

Xia K, Wang R, Ou X, Fang Z, Tian C, Duan J, Wang Y, Zhang M (2012) OsTIR1 and OsAFB2 downregulation via OsmiR393 overexpression leads to more tillers, early flowering and less tolerance to salt and drought in rice. PLoS One 7(1): e30039

Xiang Y, Tang N, Du H, Ye H, Xiong L (2008) Characterization of OsbZIP23 as a key player of the basic leucine zipper transcription factor family for conferring abscisic acid sensitivity and salinity and drought tolerance in rice. Plant Physiol 148(4): 1938-1952

Xiong L, Yang Y (2003) Disease resistance and abiotic stress tolerance in rice are inversely modulated by an abscisic acid-inducible mitogen-activated protein kinase. Plant Cell 15(3): 745-759

Yang A, Dai X, Zhang WH (2012) A R2R3-type MYB gene, OsMYB2, is involved in salt, cold, and dehydration tolerance in rice. J Exp Bot 63(7): 2541-2556

YAO Ming-zhe WJ-f, CHEN Hong-you, ZHAI Hu-qu, ZHANG Hong-sheng (2005) Inheritance and QTL Mapping of Salt Tolerance in Rice RICE SCI 12(1): 25-32

Zang J, Sun Y, Wang Y, Yang J, Li F, Zhou Y, Zhu L, Jessica R, Mohammadhosein F, Xu J, Li Z (2008) Dissection of genetic overlap of salt tolerance QTLs at the seedling and tillering stages using backcross introgression lines in rice. Sci China C Life Sci 51(7): 583-591

Zhang X, Long Y, Huang J, Xia J (2020) OsNAC45 is Involved in ABA Response and Salt Tolerance in Rice. Rice (N Y) 13(1): 79

Zheng X, Chen B, Lu G, Han B (2009) Overexpression of a NAC transcription factor enhances rice drought and salt tolerance. Biochem Biophys Res Commun 379(4): 985-989

Zhou YB, Liu C, Tang DY, Yan L, Wang D, Yang YZ, Gui JS, Zhao XY, Li LG, Tang XD, Yu F, Li JL, Liu LL, Zhu YH, Lin JZ, Liu XM (2018) The Receptor-Like Cytoplasmic Kinase STRK1 Phosphorylates and Activates CatC, Thereby Regulating H2O2 Homeostasis and Improving Salt Tolerance in Rice. Plant Cell 30(5): 1100-1118

Zou M, Guan Y, Ren H, Zhang F, Chen F (2008) A bZIP transcription factor, OsABI5, is involved in rice fertility and stress tolerance. Plant Mol Biol 66(6): 675-683
